# Integrated Hydraulic and Mathematical Evaluation of Flat Sheet Polyamide Reverse Osmosis Membranes for Poultry Slaughterhouse Wastewater Treatment

**DOI:** 10.3390/polym18161974

**Published:** 2026-08-13

**Authors:** Andrei Zaharia, Valentin Nedeff, Juan A. López-Ramírez, Dumitra Raducanu, Narcis Barsan, Emilian Mosnegutu

**Affiliations:** 1 Department of Environmental Engineering and Mechanical Engineering, Faculty of Engineering, “Vasile Alecsandri” University of Bacau, Calea Marasesti 156, 600115 Bacau, Romania; andrei.zaharia@ub.ro (A.Z.); narcis.barsan@ub.ro (N.B.); emos@ub.ro (E.M.); 2 The Academy of Agricultural and Forestry Sciences “Gheorghe Ionescu-Sisesti”, 61 Marasti, 011464 Bucharest, Romania; 3 Department of Environmental Technologies, Faculty of Marine and Environmental Sciences, Instituto Universitario de Investigación Marina (INMAR), Campus de Excelencia Internacional del Mar (CEIMAR), University of Cadiz, 11510 Puerto Real, Spain; juanantonio.lopez@uca.es; 4 Department of Biology, Faculty of Science, “Vasile Alecsandri” University of Bacau, 157 Calea Marasesti Street, 600115 Bacau, Romania; dora.raducanu@ub.ro

**Keywords:** polyamide membrane, life cycle, retention rate, reversible and irreversible resistance, reuse of water from poultry slaughterhouses

## Abstract

Reuse of industrial wastewater contributes to the conservation of freshwater resources and the implementation of the principles of the circular economy. In this study, the cyclic performance of a flat-sheet polyamide reverse osmosis membrane (PA-RO) for the advanced treatment of wastewater from a poultry slaughterhouse, pretreated by dissolved air flotation (DAF), was evaluated. The membrane was operated at three recirculation flow rates in successive filtration and chemical cleaning cycles to evaluate its hydraulic behavior, retention efficiency, fouling evolution, and permeate quality. Among the conditions investigated, the recirculation flow rate of 0.5 L/min provided the highest hydraulic stability and flux recovery under the investigated conditions, although hydraulic and microbiological performances were not optimized under the same operating conditions. The progressive deterioration of membrane performance during cyclic operation was associated with the accumulation of reversible and irreversible fouling, evidenced by the increase in hydraulic resistances and the incomplete flux recovery after chemical cleaning. Hierarchical clustering analysis (HCA) and mathematical modeling revealed strong relationships between operating conditions, membrane performance, and fouling evolution, generating predictive models with high coefficients of determination. The results demonstrate the potential of the cyclic operation of the PA-RO membrane to obtain a high-quality permeate intended for industrial reuse and provide a practical framework for optimizing operating conditions and fouling control strategies in membrane wastewater treatment processes.

## 1. Introduction

Within the food industry, the poultry processing sector is characterized by high specific water consumption. Effluent discharge volumes are intrinsically linked to production scales, which are driven by fluctuating consumer demand [1,2,3,4]. Poultry processing, particularly broiler slaughtering, is a water-intensive operation, with consumption rates varying significantly based on the unit operations involved and the implemented processing technologies [5].

Effluents derived from poultry processing exhibit high concentrations of complex organic loads (meat residues, feathers, blood, lipids, proteins, and antibiotics) and inorganic constituents (heavy metals, nitrogenous compounds such as nitrites and nitrates, and phosphates) [6,7,8]. These industrial streams are typically characterized by key physicochemical indicators, including turbidity, pH, total suspended solids (TSS), oil and grease (OG), chemical oxygen demand (COD), biochemical oxygen demand (BOD_5_), total phosphorus (TP), and total nitrogen (TN) [9,10,11,12]. Such a complex composition makes wastewater from poultry slaughterhouses particularly prone to membrane fouling during reverse osmosis treatment, as proteins, lipids, colloids, dissolved organic matter, and microorganisms contribute to organic fouling, biofouling, and concentration polarization [13,14,15].

Standard treatment training for poultry wastewater generally employs multi-stage configurations. Primary treatment focuses on the separation of macro-solids and coarse fractions, followed by secondary physicochemical processes, such as coagulation, flocculation, and sedimentation, often utilizing DAF or primary clarifiers. Finally, a biological stage is implemented in which microbial consortia degrade the remaining organic fraction [9,16,17,18,19,20,21,22,23,24,25]. However, despite the effective removal of suspended solids and biodegradable organic matter, residual dissolved compounds, nutrients, and microorganisms often remain in the treated effluent, requiring advanced membrane processes as a purification step [13,26].

Given the increasing stringency of European regulations regarding water reclamation, there is a growing industrial imperative to implement advanced treatment technologies. To achieve the high-quality permeate required for diverse reuse applications, conventional methods must be supplemented by advanced oxidation or membrane processes [27,28,29,30,31]. Membrane technology, particularly RO, has emerged as one of the most effective solutions for the rejection of recalcitrant pollutants [15,32,33,34,35]. RO systems can operate at elevated pressures, utilizing dense, asymmetric composite membranes capable of rejecting solutes down to a molecular level [36].

Thin-film composite (TFC) membranes with a selective polyamide (PA) layer are currently the dominant technology in the field of reverse osmosis, accounting for approximately 90% of the RO membranes available on the market. Their widespread use is due to a combination of high water permeability, excellent retention of dissolved salts and organic contaminants, good mechanical stability, and compatibility with large-scale water and wastewater treatment applications. These characteristics make commercial PA-TFC membranes the material of choice for advanced wastewater recovery and water reuse processes [37,38,39].

Despite its excellent separation efficiency, the long-term application of RO technology to wastewater from poultry slaughterhouses remains a challenge due to severe membrane fouling. Organic matter, proteins, lipids, suspended solids, and microorganisms accumulate on the membrane surface, while dissolved substances contribute to concentration polarization and, under favorable chemical conditions, to the formation of mineral deposits. These mechanisms gradually reduce the permeate flow rate, increase hydraulic resistance, decrease membrane productivity, increase the frequency of chemical cleaning, and ultimately shorten the membrane’s service life [14,15,26,40,41,42].

The degree of membrane fouling is largely determined by the hydrodynamic operating conditions, particularly the cross-flow velocity, transmembrane pressure, feed composition, and filtration duration, which influence the transport of fouling substances, concentration polarization, deposit layer formation, and the increase in hydraulic resistance [43,44].

The recent literature has explored various integrated configurations. Fatima et al. [44] demonstrated that a sequential UF-FO-RO process achieved nearly 100% removal efficiency for COD, TS, and phosphorus in poultry effluents, though TN removal remained limited to 62% due to the high solubility of ammonia [25]. Similarly, comparative studies on PVDF and polysulfone membranes have shown that smaller pore sizes (e.g., 0.05 µm) can achieve up to 91% COD retention when operated at optimal pressures [42]. Furthermore, the integration of DAF-UF-RO trains has proven capable of producing high-quality permeate suitable for non-contact reuse, such as facility sanitation, although additional polishing stages are required for direct-contact applications to further reduce COD and ammonium concentrations [45,46]. Although these studies have demonstrated excellent efficiency in removing pollutants, they have primarily evaluated the quality of the permeate and the short-term performance of the process.

Despite this progress, there is little information available on the cyclic behavior of flat-surface polyamide (PA) membranes used to treat wastewater from poultry slaughterhouses. In particular, the evolution of reversible and irreversible fouling resistances (Rrev and Rir), the flow recovery rate (FRR), the total flow reduction (TFR), and their integration into predictive mathematical models remain insufficiently studied [14,15,40,47,48].

The present study proposes an integrated evaluation of cyclic membrane performance, fouling evolution, and cleaning efficiency using a flat-sheet PA reverse osmosis membrane, treating wastewater from real poultry slaughterhouses. Unlike previous studies that mainly focused on pollutant removal efficiency, the proposed methodology combines hydraulic fouling indicators (FRR, TFR, Rrev, and Rir), pollutant retention analysis, organic matter characterization, hierarchical cluster analysis (HCA), and predictive mathematical modeling in a single experimental framework. This integrated approach provides a comprehensive understanding of membrane behavior under realistic operating conditions and contributes to the identification of mechanisms governing long-term membrane performance and fouling development.

The objective of this study was to evaluate the cyclic hydraulic and separation performance of a polyamide (PA) reverse osmosis membrane used to treat actual wastewater from a poultry slaughterhouse, as well as to determine the influence of hydrodynamic operating conditions on permeate flow, fouling behavior, hydraulic resistance, membrane efficiency, and permeate quality.

## 2. Materials and Methods

### 2.1. Characteristics of Wastewater Analyzed in the Study

**Figure 1:**
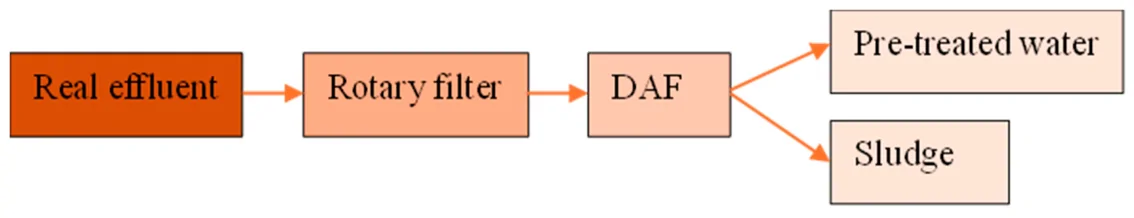
Pretreatment stages applied in the poultry slaughterhouse.

The slaughterhouse is designed for a maximum processing capacity of 160 metric tons of chicken carcasses per day. This capacity is based on the number of live birds intended for slaughter and on an average slaughter yield of approximately 60% under normal operating conditions [49]. Both the slaughtering and treatment processes have been described in detail in the paper entitled ‘Some practical approaches of the poultry slaughter wastewater treatment by apply physicochemical treatment’ [50]. Samples were taken from the wastewater after the DAF process at the poultry slaughterhouse’s wastewater treatment plant. Wastewater samples were collected during the first part of each experimental day. The relevance of the sampling is ensured by its alignment with the intensity of the slaughtering process and with the operating regime of the treatment plant. The slaughterhouse wastewater treatment plant consists of the stages shown in Figure 1.

The DAF process is carried out using 0.05% polymeric flocculant solution (2.5 kg/day), a 42% FeCl_3_ (25 L/h), and a 25% NaOH solution (18 L/h). The DAF process is operated under the following conditions: pH of 6.4, a maximum pressure of 5.2 bar, an average flow rate of 110 m^3^/h, and compressed air was introduced at regular intervals (30 min).

Samples were taken every day of the experiment; therefore, Table 1 shows the ranges and mean values of the quality indicators of the raw wastewater.

### 2.2. Methods of Determination

All physical, chemical, and biological quality indicators were determined using laboratory equipment after each treatment stage applied in the treatment train. Conductivity was determined using the COND 3210 WTW portable device (Wissenschaftlich-Technische Werkstätten GmbH, Weilheim, Germany), while pH and turbidity were determined using the pH Meter 3210 WTW (Wissenschaftlich-Technische Werkstätten GmbH, Weilheim, Germany) and Turb 430 IR (WTW/Xylem Analytics Germany Sales GmbH & Co. KG, Weilheim, Germany), respectively. COD and TOC were determined using the DR3900 spectrophotometer (Hach Lange GmbH, Düsseldorf, Germany) and the LT200 digital themoreactor (Hach Lange GmbH, Düsseldorf, Germany), using analysis kits provided by Hach, Düsseldorf, Germany. TSS was determined by the photometric method using rectangular cuvettes. Heterotrophic plate counts (HPC) were determined according to the standard procedure ISO 6222:1999 [51]. The colony-forming unit method was used to evaluate the efficiency of the PA membrane in removing microorganisms. This method is based on the number of colonies that develop after inoculating 1 mL of the analyzed sample onto a standard culture medium (PCA) after incubation at 37 °C for 24–48 h [34].

### 2.3. Experimental

**Figure 2:**
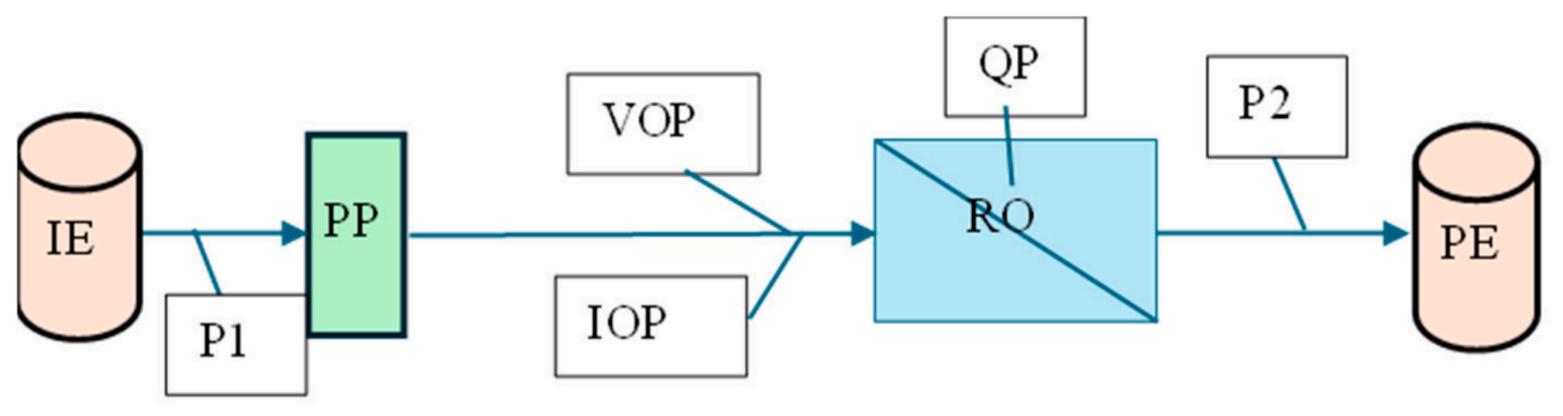
Experimental protocol, including the main RO stage. IE—Industrial Effluent; PP—Polypropylene filter; RO—Reverse Osmosis; PE—Purified effluent; P1 and P2—sampling points IOP—Invariable operating parameters (effluent volume; temperature, etc.); VOP—Variable operating parameters (inlet pressure and flow rate); QP—Quality parameters determined during the RO process (pH, conductivity, turbidity, temperature, permeate volume).

This study focused on analyzing the RO process, so the performance of the RO process was determined comparatively for both pure water and real effluents subjected to different operating pressures and flow rates. The experiments were organized according to the experimental process diagram shown in Figure 2.

The experimental protocol was organized according to a three-factor design, comprising nine experiments, to which three repetitions were added for data accuracy.

#### 2.3.1. Description of the RO Process in Treating Wastewater from Poultry Slaughterhouses

**Figure 3:**
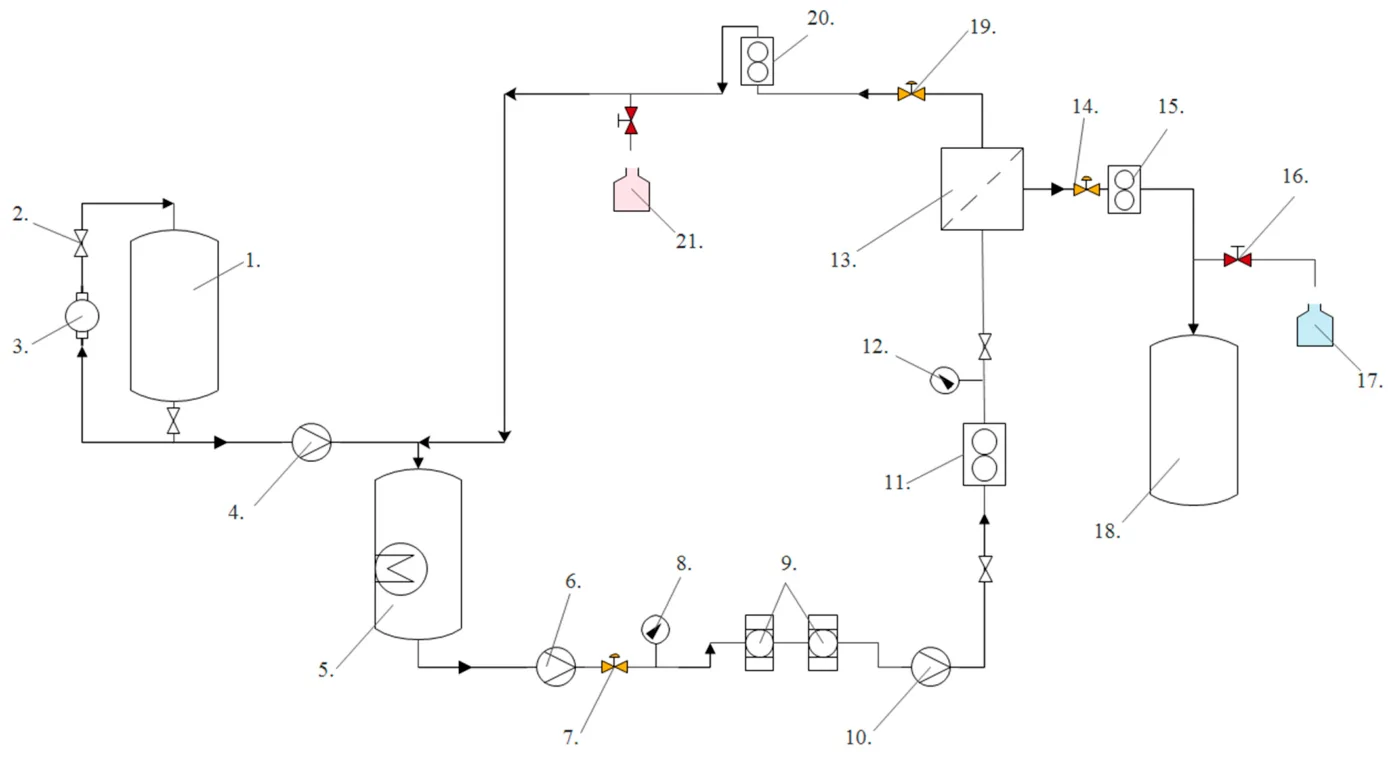
Process diagram of the RO plant with PA flat membrane. 1. Pretreated effluent intake tank. 2. Control valve. 3. Recirculation pump. 4. Intake pump; 5. Pretreated effluent tank. 6. Intake pump. 7. Pressure control valve. 8. Manometer. 9. Polypropylene prefilter. 10. Feed pump. 11. Flow meter. 12. Barometer with clock. 13. Flat module with RO polymer membrane frame. 14. Permeate flow meter control valve. 15. Permeate flow meter. 16. Permeate sampling tap. 17. Permeate container. 18. Permeate tank. 19. Concentrate flow meter control valve. 20. Concentrate flow meter. 21. Concentrate container.

The reverse osmosis experiments were conducted using a commercially available thin-film composite (TFC) membrane (Vontron ULP1812, Vontron Technology Co., Ltd., Guiyang, China) consisting of an active layer of aromatic polyamide supported by a porous polysulfone substrate. The membrane was mounted in a plate-and-frame module with an effective filtration area of 0.0161 m^2^ and an overall thickness of 0.12 mm, including the active polyamide layer and its support structure. According to the manufacturer’s specifications, the membrane has an approximate pore size of 0.0001 μm, an operating pH range of 6.5–8.5, a recommended operating temperature of 25 °C, a feed flow rate of 120–250 L h, and a maximum operating pressure of 8.5 bar. Figure 3, illustrates the process diagram of the RO pilot plant with its main components.

For each experiment, the feed tank of the reverse osmosis system was filled with 10 L of wastewater pretreated by DAF. This volume was recirculated during three successive filtration cycles. The samples were stored at 4 °C prior to use. The quality of the wastewater that underwent the pretreatment process at the poultry slaughterhouse was evaluated.

Before being fed into the reverse osmosis module, the effluent treated by DAF was diluted 1:1 (*v*/*v*) with distilled water and passed through a cartridge-type prefilter.

The membrane module is operated at a constant pressure of 6 bar in cross-flow mode with recirculation at flow rates of 0.5, 1.5, and 3 L/min. The operating pressure of 6 bar was selected to evaluate the performance of the commercial polyamide ultra-low pressure reverse osmosis membrane under low pressure operating conditions. Thermal stability was ensured by a coil heat exchanger located in the inlet tank of the plant.

The concentrate was recirculated within the plant, and the permeate was collected separately for quantitative and qualitative analysis. The experimental protocol consisted of successive cycles of 180 min each, with a total time of 540 min per experiment. Every 30 min, the permeate volume was determined and, at the same time, the quality indicators were analyzed in order to monitor the performance dynamics during each cycle and for each hydraulic regime. The membrane was replaced at the beginning of each experiment to ensure reproducible initial conditions. The evaluation focused on the stability of operation at 6 bar, in terms of the influence of the cross-flow velocity on the concentration polarization layer and on the ability of the membrane system, to generate a permeate that complies with the limits permitted for reuse.

#### 2.3.2. Protocol for Cleaning the PA Membrane

The cleaning protocol was performed after each filtration cycle with real effluent from a poultry slaughterhouse. Since the PA membrane was exposed to high concentrations of organic compounds and microorganisms, cleaning was performed with 0.1% (*w*/*w*) NaOH and 0.1% (*w*/*w*) EDTA solutions at an optimal pH of 12. Cleaning was performed by immersing the flat membrane in a covered container placed on a vibrating table. The cleaning solution was diluted using pure (deionized) water. The cleaning time was 30 min, and before and after this stage, the membrane was rinsed with pure water. After the cleaning stage, the membrane was immersed in a solution of sodium metabisulfite and pure water to ensure its preservation until the next filtration cycle.

### 2.4. Statistical Determination

The data obtained were processed and analyzed using equations that described the efficiency of the membrane in reducing pollutant compounds in the effluent, the degree of polarization/membrane fouling, and the flux recovery of the RO membrane.

The membrane’s capacity to retain pollutants from real effluent is evaluated using Equation (1) [52]:

(1) $$E_{f}=\left(1-\frac{A_{p}}{A_{i}}\right)\times 100$$

where *E_f_*—Retention efficiency; *A_p_*—Concentration of polluting compounds in the permeate; *A_i_*—Concentration of initial polluting compounds.

Productivity and membrane fouling are defined by the membrane flux, which was calculated using Equation (2) [52]:

(2) $$J=\frac{V}{A\times t}$$

where *J* is the membrane flux (L/m^2^h); *V*—Permeate volume (L); *A*—Active membrane surface area (m^2^); *t*—Effective filtration time (h):

In order to determine the integrity and performance of the membrane, the Flow Recovery Rate was determined using Equation (3) [53]:

(3) $$FRR=\left(\frac{J_{w2}}{J_{w1}}\right)\times 100$$

where *J*_*w*2_ is the flow determined with pure water after the cleaning process; *J*_*w*1_ —is the initial flow determined with pure water before reusing the membrane.

The Total Flow Reduction Ratio (*TFR*) quantifies the influence of fouling on membrane flow and is defined according to Equation (4). After the filtration process, at a frequency of 10 min for 30 min, the permeate volume was collected to determine the TFR relationship [54].

(4) $$TFR=\frac{J_{w1}-J_{w3}}{J_{w1}}$$

where *J*_*w*1_ represents the initial flow determined with pure water before using the membrane in the filtration process (L/m^2^h); *J*_*w*3_—the flow determined with pure water after the filtration process (L/m^2^h).

Fouling resistance is composed of the sum of reversible and irreversible resistance given by Darcy’s Law.

Irreversible resistance suggests permanent clogging and polarization of the membrane pores and is defined by Equation (5). This equation was used to compare the membrane’s ability to recover after the cleaning process determined between the three filtration cycles [55].

(5) $$R_{ir}=\frac{∆P}{\mu \times J_{1}\;}-\frac{∆P}{\mu \times J_{0}}$$

where *R_ir_* represents irreversible resistance (m^−1^); Δ*P*—operating pressure (bar); µ—viscosity of distilled water (Pa·s); *J*_0_—initial average flow of the clean membrane (L/m^2^h); *J*_1_—flow determined after the cleaning process (L/m^2^h).

Reversible resistance represents the possibility for the membrane to recover using hydraulic cleaning processes without affecting the structure of the membrane material. Equation (6) describes reversible resistance [55]:

(6) $$R_{rev}=\frac{∆P}{\mu \times J_{2}\;}-\frac{∆P}{\mu \times J_{1}}$$

where *Rrev* represents reversible resistance; Δ*P*—operating pressure (bars); *µ*—viscosity of distilled water (Pa·s); *J*_2_—flow determined at the end of the filtration process with real effluent (L/m^2^h); *J*_1_—flow determined after the cleaning process (L/m^2^h).

Total fouling resistance (*Rf*) was determined to quantify the total hydraulic resistance generated by the accumulation of deposits during membrane filtration [55].

(7) $$R_{f}=R_{rev}+R_{ir}$$

## 3. Results

### 3.1. Influence of Operating Conditions on Flux

**Figure 4:**
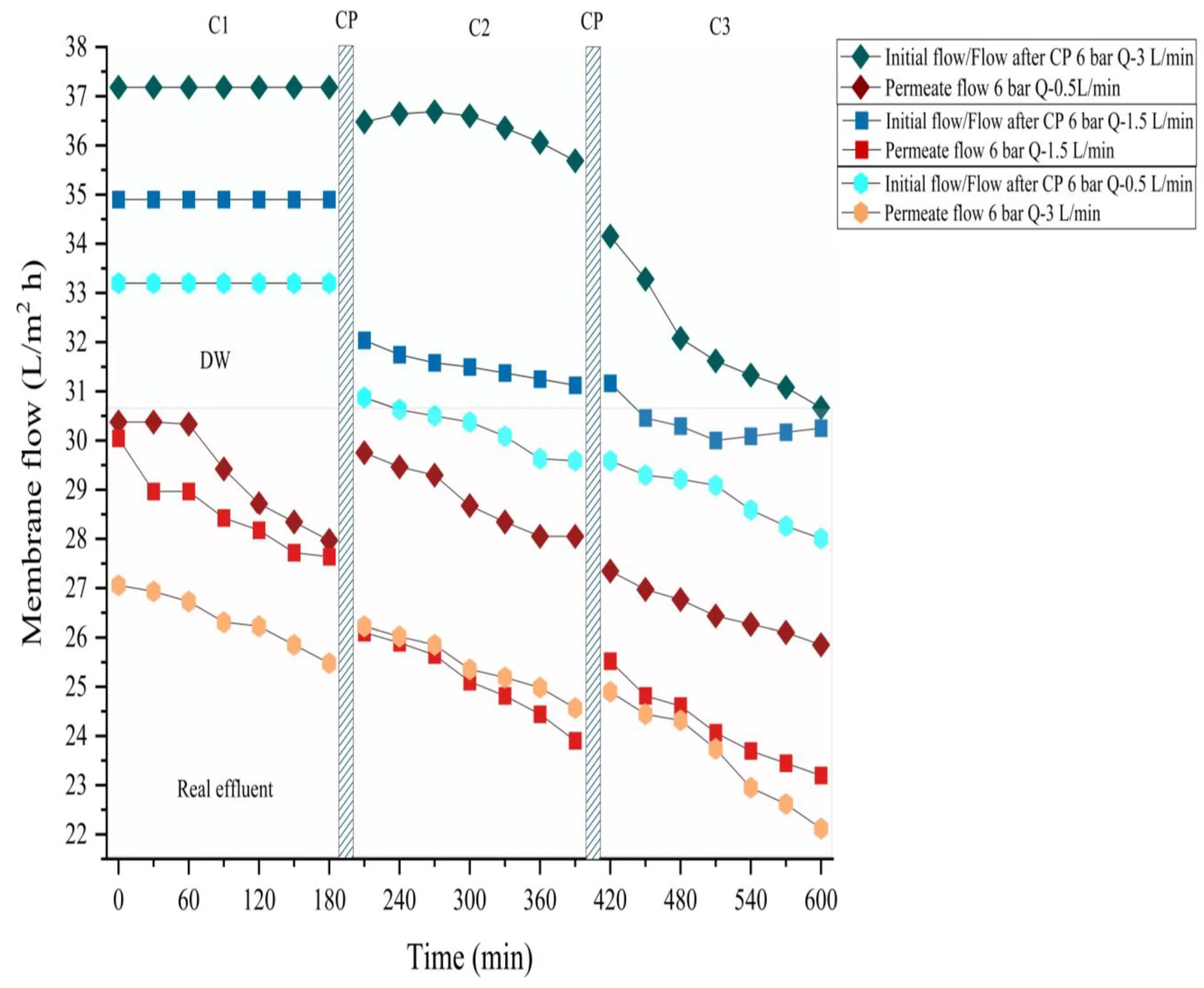
Influence of operating conditions on flux during the three filtration cycles with the PA membrane.

Figure 4 illustrates the evolution of the permeate flux during cyclic filtration of real effluent from a slaughterhouse using a flat-sheet PA-RO membrane operating at a pressure of 6 bar and cross-flow rates of 0.5, 1.5, and 3 L/min. Under all investigated conditions, the permeate flux showed a progressive decrease both during each filtration cycle and between successive cycles C1–C3, indicating the gradual development of fouling phenomena and an increase in hydraulic resistance during repeated membrane operation.

For all investigated conditions, the permeate flux showed a progressive decline both within individual cycles and between successive C1–C3 cycles, indicating the gradual development of fouling mechanisms and the accumulation of hydraulic resistance during repeated membrane operation.

The flux measurements conducted using distilled water revealed a gradual decrease in membrane permeability after each filtration and cleaning step.

At a flow rate of 0.5 L/min, the initial flux decreased from 37.18 L/m^2^h in C1 to approximately 29.59 L/m^2^h in C3, indicating the gradual accumulation of an irreversible fouling component during successive operation.

Similar trends were observed at flow rates of 1.5 and 3 L/min, but the reduction in flux became more pronounced in the later filtration cycles.

For the real effluent, the permeate flow rate showed a continuous decline over the 180 min of operation for all conditions investigated.

At a flow rate of 0.5 L/min, the flux rate reductions were relatively moderate within each filtration cycle, suggesting the predominance of reversible mechanisms associated with concentration polarization and the accumulation of a superficial layer of deposits.

In contrast, at flow rates of 1.5 and 3 L/min, the decline in flux became more pronounced, particularly in cycles C2 and C3, indicating the progressive development of an irreversible fouling component.

The increase in cross-flow rate intensified the shear forces and contributed to a partial limitation of concentration polarization, but did not lead to a proportional increase in permeate flux. This behavior suggests that the membrane’s hydraulic performance was simultaneously influenced by mass transfer, the resistance of the fouling layer, and changes in the hydrodynamic conditions within the membrane module’s flow channel.

In the case of the real effluent from the poultry slaughterhouse, characterized by a high organic and colloidal load, increasing the recirculation rate may promote the compaction of the previously formed fouling layer and an increase in the hydraulic resistance associated with water transfer through the membrane.

The incomplete recovery of flux after successive CP confirms the gradual accumulation of irreversible resistance during cyclic operation.

Thus, the evolution of the permeate flux highlights that the performance of the PA-RO membrane is controlled by the interaction between concentration polarization, the development of the fouling layer, and the progressive accumulation of irreversible clogging components during filtration.

### 3.2. Influence of Operating Conditions on Conductivity and pH

**Figure 5:**
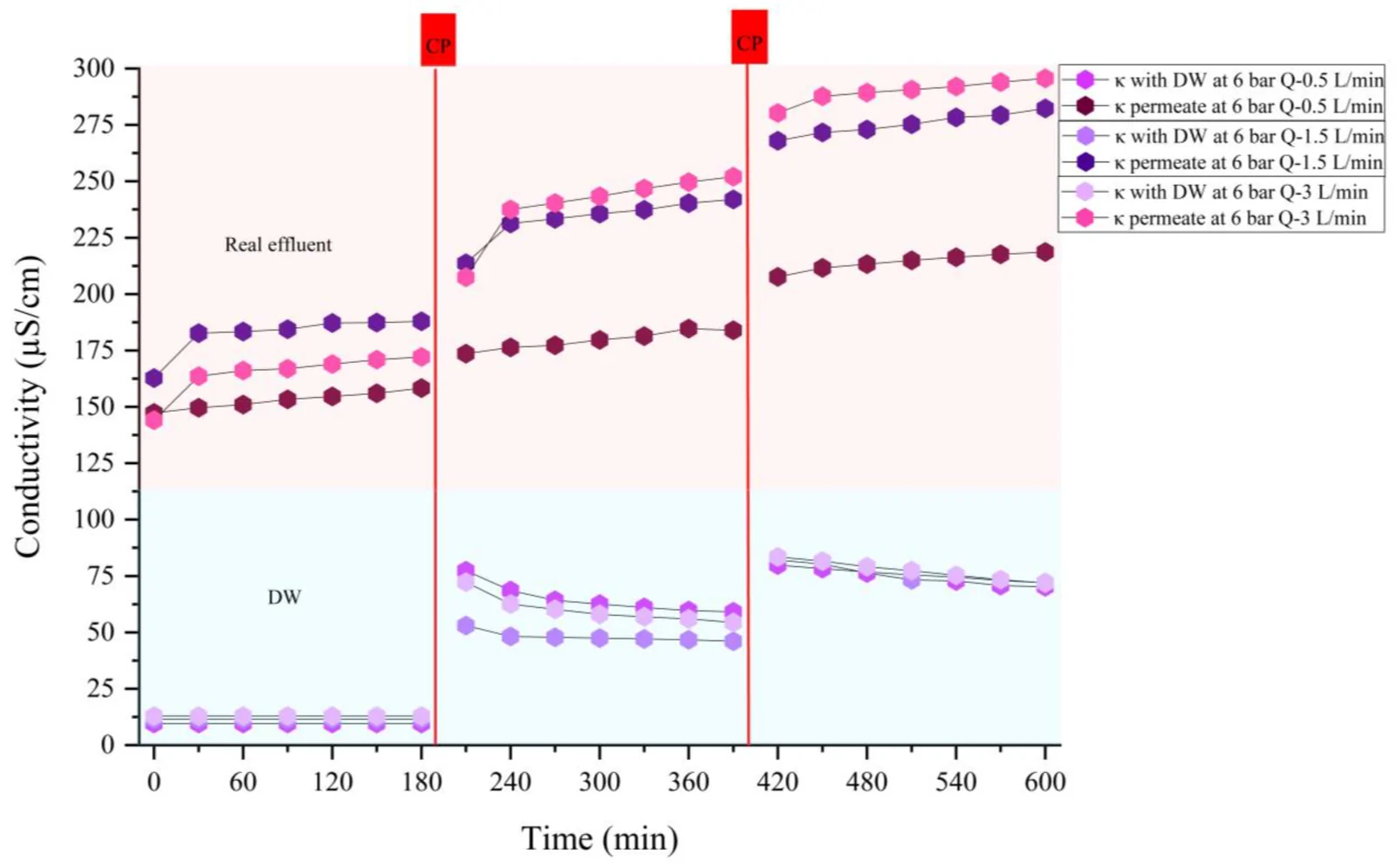
Conductivity variation during the three filtration cycles with the PA membrane, determined for both DW and real effluent.

Figure 5 shows the average variations in permeate conductivity determined for both DW and real effluents resulting from the three PA membrane filtration cycles at a pressure of 6 bar and flow rates of 0.5, 1.5, and 3 L/min.

The conductivity of the permeate obtained by RO was directly influenced by the hydrodynamic conditions and the progression of fouling phenomena that developed during the filtration of real effluent from the poultry slaughterhouse.

At a pressure of 6 bar, the lowest conductivity values were obtained at a flow rate of 0.5 L/min, indicating superior ion retention and greater stability of the membrane separation mechanism.

Under these conditions, the low flow rate limited the convective transport of ions to the membrane surface and favored the maintenance of the selectivity of the polyamide active layer.

The progressive increase in conductivity from C1 to C3 highlights the gradual accumulation of organic and inorganic compounds near the membrane surface and the development of irreversible fouling phenomena.

The formation of the fouling layer and the intensification of concentration polarization favored an increase in the local concentration of salts at the membrane surface.

The persistence of these increases after applying the cleaning protocol indicates the stable adsorption of certain organic and mineral compounds within the structure of the membrane’s active layer.

At flow rates of 1.5 and 3 L/min, the conductivity values were higher compared to the flow rate of 0.5 L/min, especially in C2 and C3. Although the increase in flow rate can partially reduce the thickness of the boundary layer, it simultaneously intensifies the convective transport of ionic species to the membrane surface.

In the case of real effluent, characterized by a high organic and saline load, this phenomenon contributed to the decrease in membrane selectivity and the increase in the concentration of dissolved ions in the permeate.

The progressive decrease in the conductivity reduction efficiency confirms the gradual degradation of the membrane performance during successive operations on real effluent.

The continuous interaction between the compounds present in the wastewater and the active surface of the membrane affected the electrochemical mechanisms of ionic rejection, contributing to the increased permeability of dissolved species and the decrease in the overall desalination efficiency.

**Figure 6:**
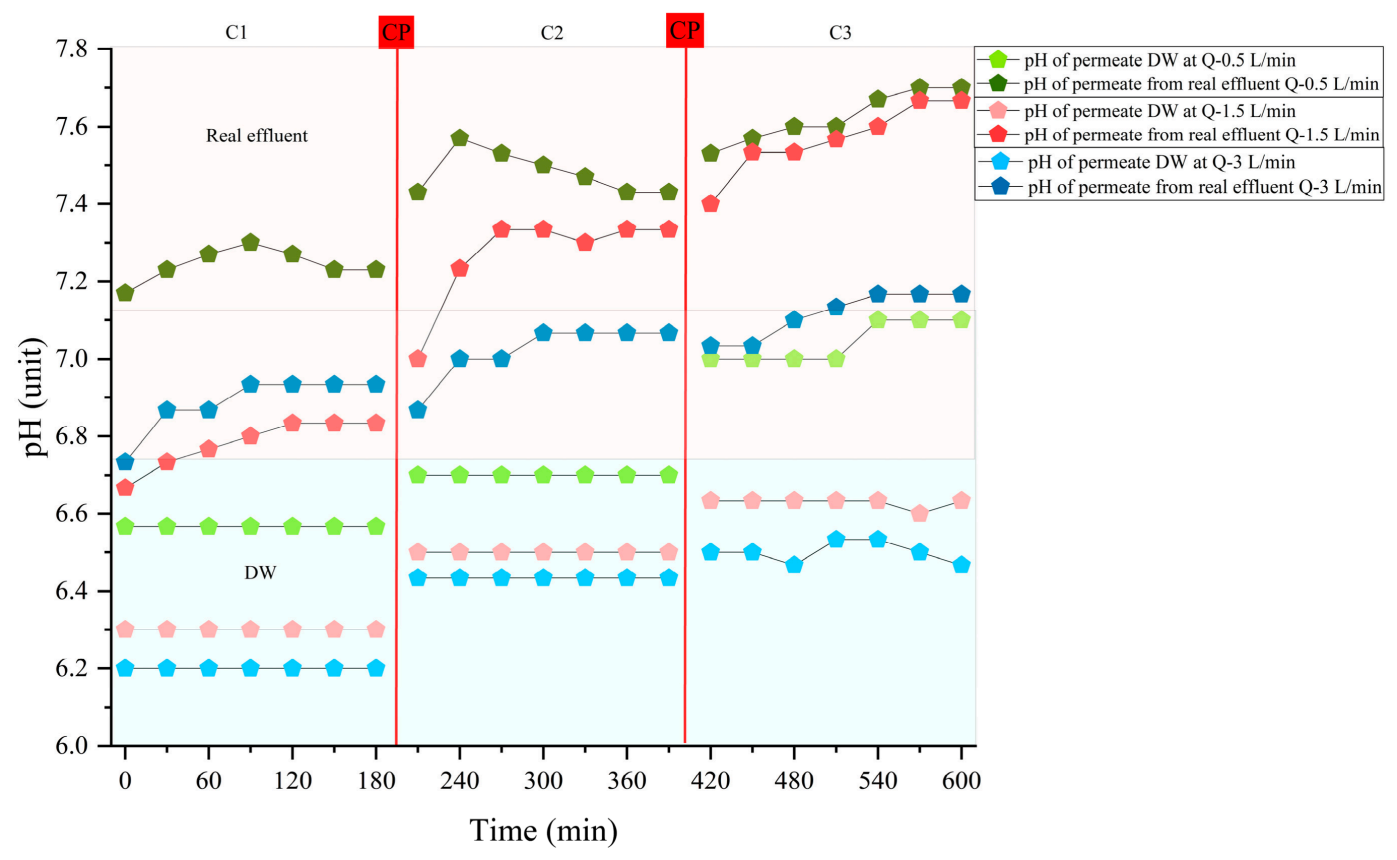
Average pH variation during the three filtration cycles of DW and real effluent with PA membrane from RO at a pressure of 6 bar, applied at flow rates of 0.5, 1.5, and 3 L/min, with a total volume of 10 L and a constant temperature of 25 °C.

Figure 6 shows the average pH variation determined during the three filtration cycles with DW and real effluent using the PA RO membrane.

The gradual increase in pH, observed for both DW and real effluent, indicates a progressive loss of the PA membrane’s ability to retain weakly ionized basic species, such as ammonia and bicarbonate.

This trend, accentuated in C2 and C3, reflects the apparent loss of separation performance and the modification of the chemical equilibrium at the membrane–matrix interface of the real effluent, favoring the diffusion of basic species and leading to a slight alkalization of the permeate.

### 3.3. Determination of PA Membrane Efficiency

#### Evolution of pH and Conductivity as a Function of the Filtration Cycle Applied at 6 Bar and Flow Rates of 0.5, 1.5, and 3 L/Min

**Figure 7:**
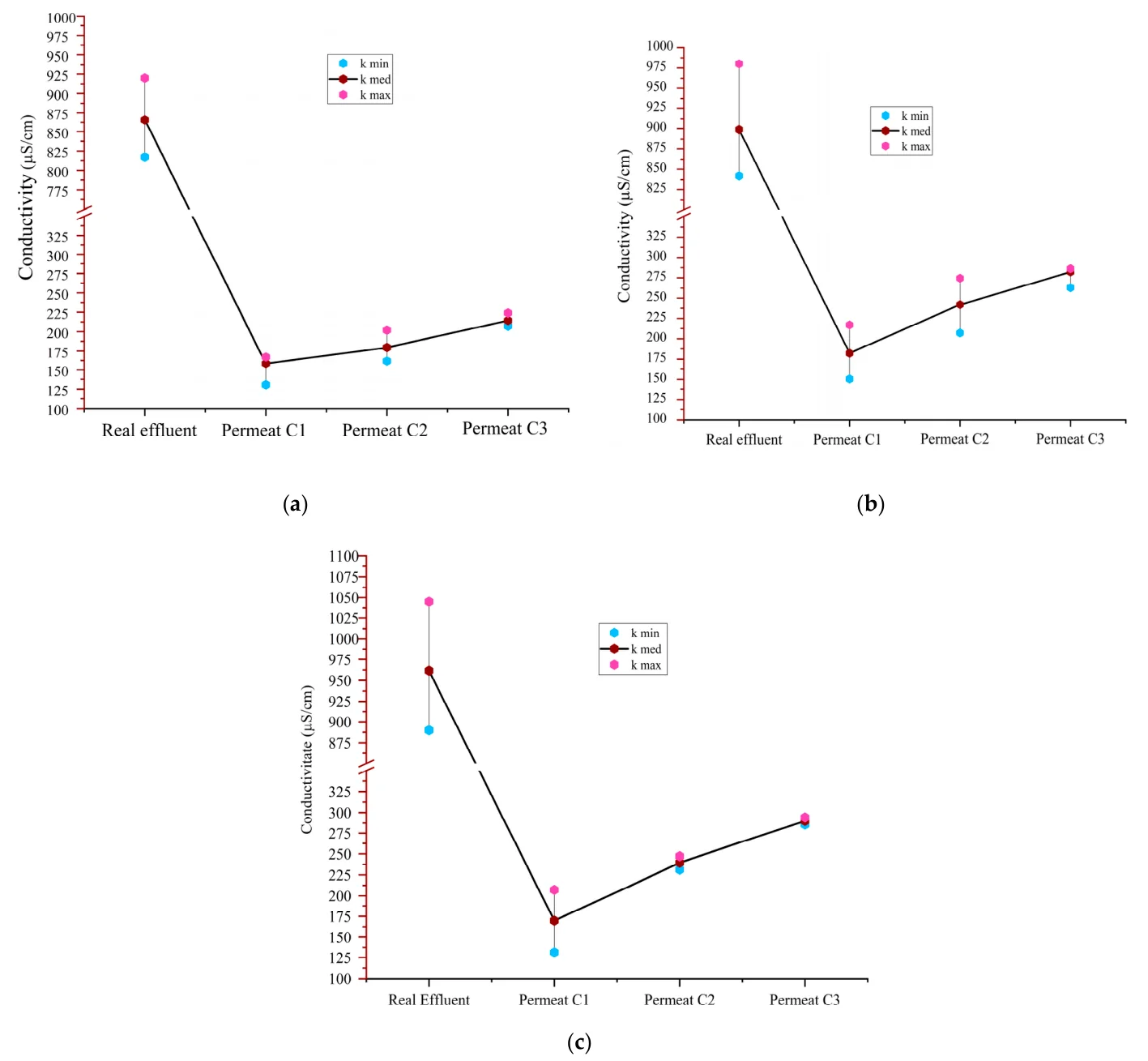
Average variation in conductivity of the actual effluent and permeate obtained from the three filtration cycles, determined at 6 bar with flow rates of (**a**) 0.5 L/min, (**b**) 1.5 L/min, and (**c**) 3 L/min.

Figure 7 shows the variations in conductivity and pH for both the actual unfiltered effluent and the permeate obtained from the three cycles following the RO process with the PA membrane, at 6 bar, tested at flow rates of 0.5, 1.5, and 3 L/min.

The progressive decrease in the degree of ion rejection observed during cyclic operation occurred simultaneously with the reduction in flux recovery and the increase in reversible and irreversible hydraulic resistances. This clearly demonstrates that the membrane performance became progressively controlled by fouling, rather than exclusively by the intrinsic selectivity of the polyamide active layer.

The increase in hydraulic resistance favored concentration polarization, reducing the effective degree of rejection of dissolved ionic species and leading to a gradual increase in permeate conductivity, observed during cycles C2 and C3.

The persistence of this behavior after chemical cleaning indicates that residual fouling substances remained attached to the membrane surface, progressively limiting the recovery of the initial separation performance.

These results demonstrate that the long-term separation performance of PA-RO membranes is determined by the combined effects of hydrodynamic conditions, fouling evolution, and cleaning efficiency, highlighting the importance of controlling these parameters to ensure stable operation under industrial conditions.

**Figure 8:**
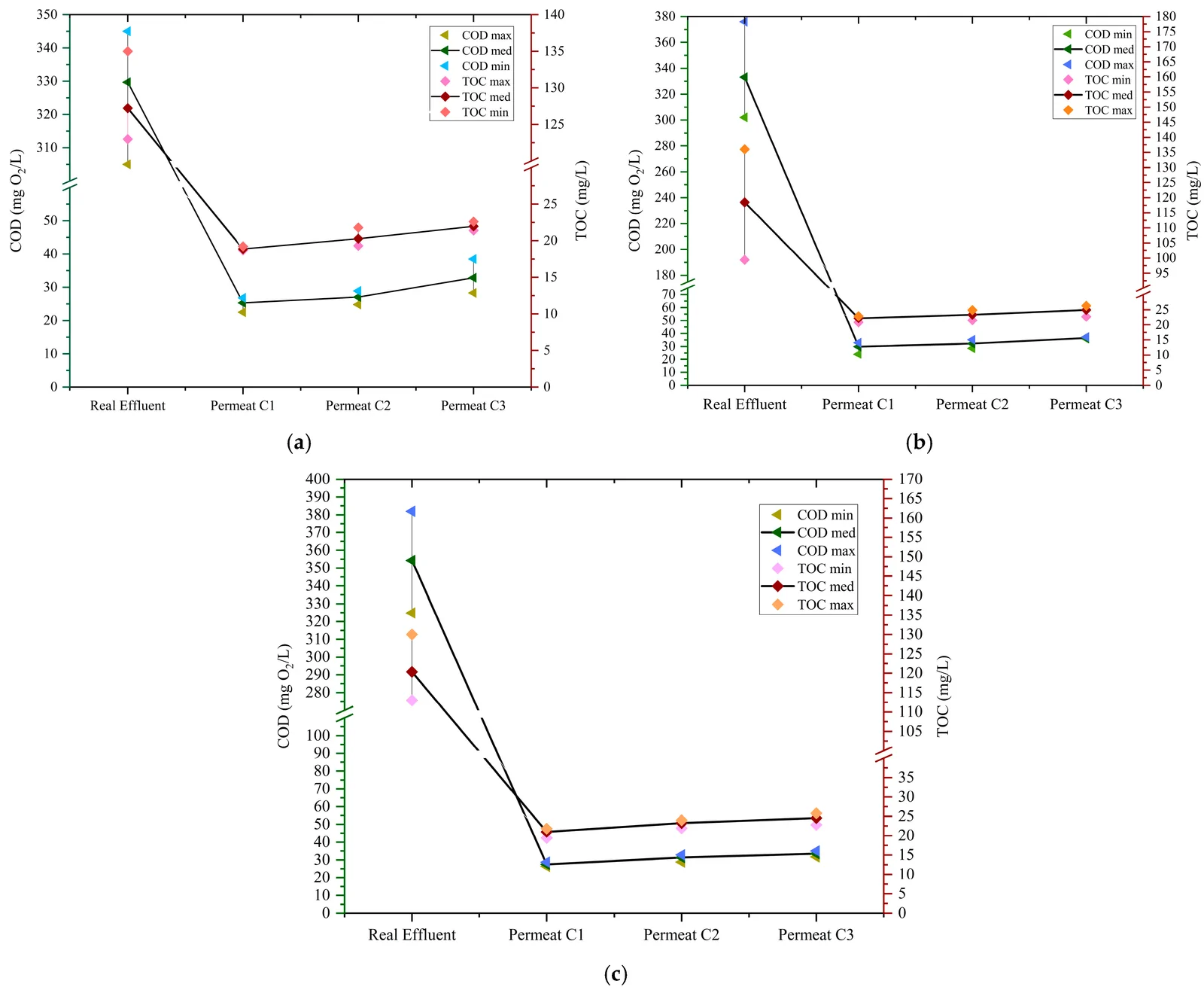
Average variation in COD and TOC was determined for the actual effluent and for the permeate obtained from the three filtration cycles, determined at 6 bar with flow rates of (**a**) 0.5 L/min, (**b**) 1.5 L/min, and (**c**) 3 L/min.

Figure 8 shows the variation in COD and TOC for both the actual effluent from a poultry slaughterhouse and the permeate obtained after the RO membrane process performed using a PA membrane at an operating pressure of 6 bar and a flow rate of 0.5 L/min.

Organic fouling progressively became the dominant mechanism governing the long-term separation performance of the PA reverse osmosis membrane during cyclic operation. This interpretation is supported by the simultaneous decrease in flux recovery and the increase in reversible and irreversible hydraulic resistances, indicating that the chemical cleaning protocol failed to completely remove the residual organic fouling accumulated during repeated filtration cycles.

The persistence of residual fouling progressively increased the hydraulic resistance and promoted concentration polarization, reducing the effective rejection of dissolved organic compounds. The applied hydrodynamic conditions determined the balance between fouling accumulation and its removal from the membrane surface. At a recirculation flow rate of 0.5 L min^−1^, fouling development remained limited, resulting in the highest and most stable COD and TOC rejection during cyclic operation. At 1.5 L min^−1^, the balance between fouling deposition and cross-flow transport maintained stable membrane performance, while at 3 L min the progressive deterioration of organic matter rejection indicated that the applied hydrodynamic conditions were no longer sufficient to counteract fouling development during prolonged operation.

The gradual deterioration of organic matter rejection is consistent with the accumulation of a residual fouling layer that persisted after chemical cleaning and progressively impaired membrane separation. This interpretation is corroborated by the evolution of FRR, TFR, and reversible and irreversible hydraulic resistances, which collectively demonstrate that membrane performance was increasingly controlled by fouling rather than by the intrinsic separation characteristics of the polyamide membrane.

These findings demonstrate that the removal of dissolved organic compounds is primarily governed by the interaction between hydrodynamic conditions, fouling development, and cleaning efficiency. Therefore, optimizing the recirculation flow rate and fouling control strategies is essential for maintaining long-term stable membrane performance in poultry slaughterhouse wastewater treatment.

**Figure 9:**
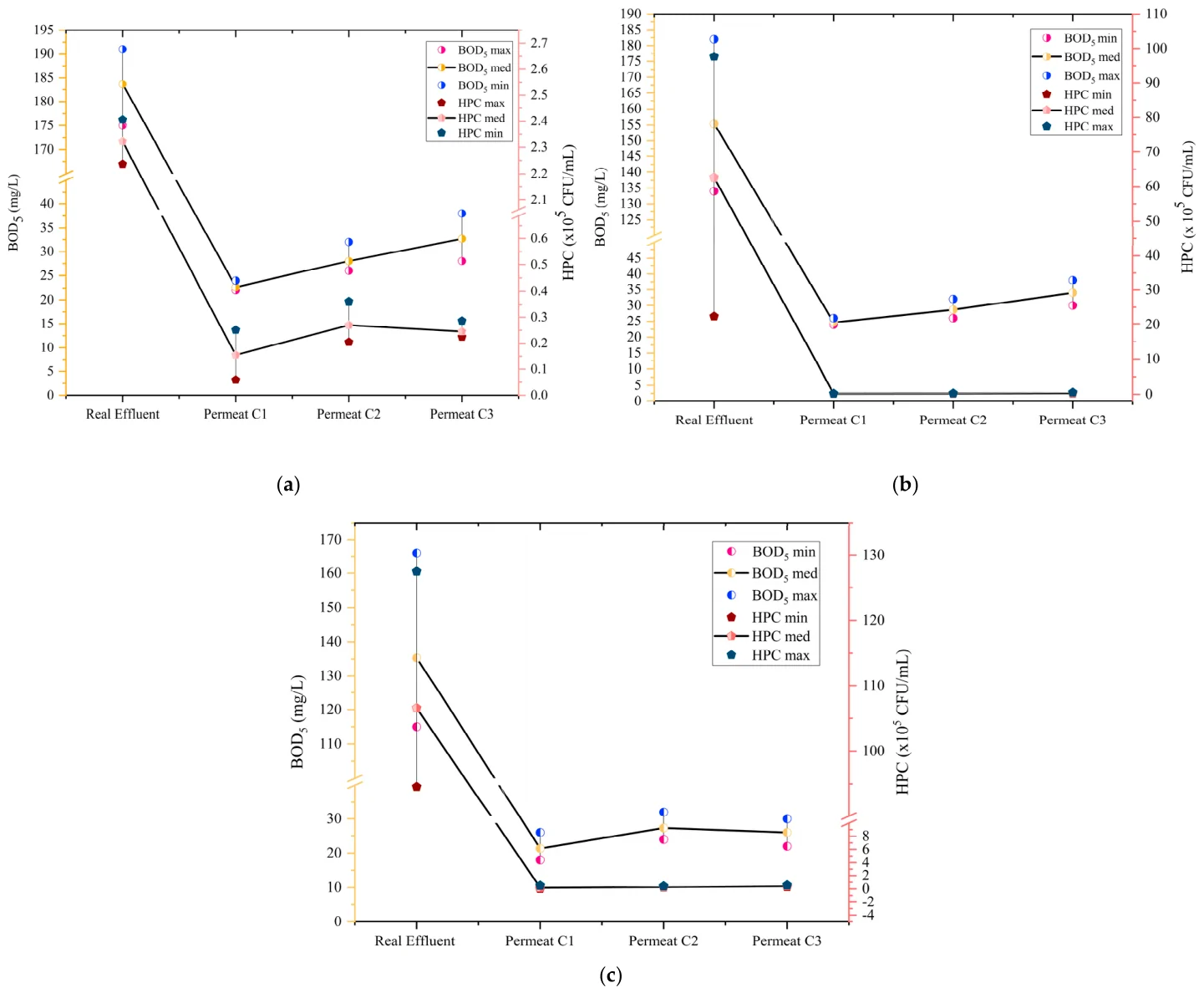
Average variation in BOD_5_ and HPC concentrations determined for actual effluents and permeate obtained from the RO process using the PA membrane at an operating pressure of 6 bar with flow rates of (**a**) 0.5 L/min, (**b**) 1.5 L/min, and (**c**) 3 L/min.

The evolution of the retention of biodegradable organic compounds and microorganisms during the cyclic operation of the reverse osmosis process is shown in Figure 9 and indicates that the fouling phenomenon influenced the dissolved organic fractions and microbiological contaminants differently through distinct separation mechanisms. While the retention of biodegradable organic compounds progressively deteriorated with the development of fouling, the retention of microorganisms remained relatively stable, consistent with the fact that steric exclusion remained the main mechanism responsible for their retention throughout the entire filtration process.

The progressive accumulation of residual organic compounds after each filtration cycle resulted in the simultaneous increase in reversible and irreversible hydraulic resistances and the reduction in flux recovery degree, indicating that CP did not completely restore the initial hydraulic performance of the membrane. Under these conditions, the residual fouling layer progressively modified the local mass transfer conditions at the membrane’s surface, decreasing the retention efficiency of biodegradable organic compounds in successive filtration cycles without significantly influencing the retention of microorganisms.

The hydrodynamic conditions controlled the rate of fouling development during repeated operation. The operating regime corresponding to the recirculation flow rate of 0.5 L/min delayed the accumulation of residual compounds and maintained the separation performance of the membrane during successive cycles. In contrast, increasing the recirculation flow rate accelerated the cumulative effects of fouling, indicating that the intensification of the flow regime was not sufficient to limit the accumulation of residual deposits after the application of CP.

This behavior highlights the fact that the long-term stability of the PA membrane used for the treatment of wastewater from poultry slaughterhouses is mainly controlled by the interaction between the evolution of fouling and the hydrodynamic operating conditions. Consequently, maintaining stable process performance requires not only efficient pretreatment steps, but also optimized cleaning strategies capable of limiting the progressive accumulation of residual compounds during cyclic operation.

### 3.4. Study of Different Organic Matter Correlations

Table 2 presents different correlations for organic matter indicators that provide an overview of the organic load and the change in the nature of organic matter after applying the RO process.

The evolution of the BOD_5_/COD and COD/BOD_5_ ratios provides information on the transformation of organic matter during the treatment process. In the raw slaughterhouse effluent, moderate BOD_5_/COD values (0.38–0.56) and high COD/BOD_5_ ratios (1.8–2.6) reflect the presence of a complex organic matrix with a partially refractory character, comprising proteins, lipids, colloids and suspended solids. Following the RO process, the BOD_5_/COD ratio increased (0.78–0.94), while the COD/BOD_5_ ratio approached unity (1.07–1.29), indicating a significant reduction in the inert or non-biodegradable fraction. This trend suggests that the membrane effectively removed macromolecular and colloidal components, resulting in a simplified, more easily biodegradable organic load in the permeate. These findings are consistent with data from the literature and confirm the ability of the PA membrane to enhance effluent biodegradability by selectively retaining complex compounds.

In the case of the COD/TOC ratio, the amount of oxygen required for chemical oxidation is expressed in relation to the unit of organic carbon and provides information about the structural complexity and the degree of oxidation of organic matter. The increased COD/TOC values in the real effluent, namely 2.59–2.94, indicate the presence of partially refractory and colloidal compounds, which require a large amount of chemical oxygen for degradation. After the RO membrane process, this ratio decreased significantly, reaching values between 1.30 and 1.46. This trend reflects the structural simplification of the organic fraction, with residual organic carbon being associated with micromolecular compounds that are easily chemically oxidizable.

The BOD_5_/TOC ratio indicates the proportion of organic carbon that is accessible to biological processes and reflects the degree of biological activity associated with the organic fraction. In the real effluent, average values above 1 suggest a significant amount of biologically active carbon, but distributed in a complex matrix that also includes poorly biodegradable fractions. In the BOD_5_/TOC ratio determined for the permeate, the values vary between 1.0 and 1.36. These trends indicate that most of the residual organic carbon contributes to the biochemical oxygen demand. Maintaining the ratio at similar values in the permeate suggests a relatively stable composition of the residual organic fraction during the three filtration cycles.

### 3.5. Membrane Performance

**Figure 10:**
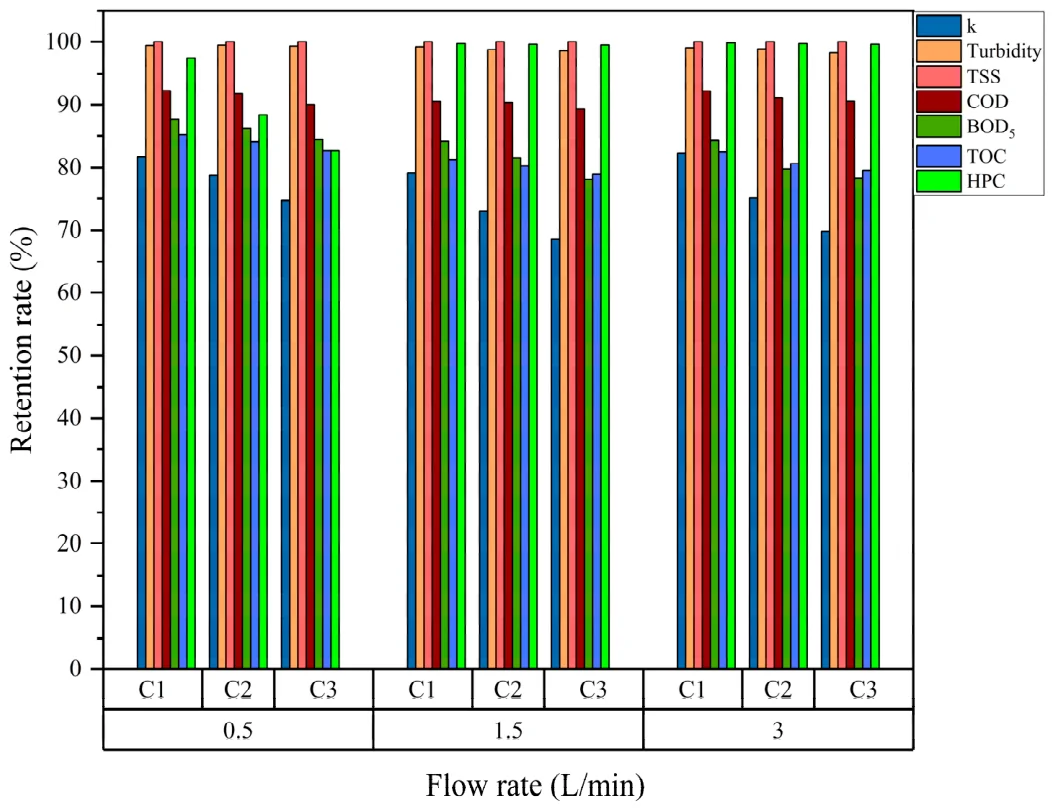
Retention rate of quality indicators determined after each filtration cycle with the PA membrane of RO at a pressure of 6 bar with different flow rates (0.5, 1.5 and 3 L/min).

Figure 10 shows the variation in the retention ratio of the membrane determined for the quality indicators of the actual effluent obtained from the three filtration cycles tested at operating flow rates. These graphs show the differentiated analysis of the membrane performance.

Retention rates showed a constant trend for turbidity and TSS, confirming the predominance of size-exclusion mechanisms for colloidal particles and suspended solids, independent of the recirculation flow rate.

In contrast, variations in conductivity retention were driven by changes in mass transfer and concentration polarization at the interface between the membrane and the real effluent. At flow rates of 1.5 and 3 L/min, the intensification of tangential flow reduced the boundary layer thickness and favored the transport of ionic species toward the membrane surface, leading to a progressive decrease in conductivity retention in cycles C2 and C3.

Maintaining HPC retention above 99% at flow rates of 1.5 and 3 L/min demonstrates that microbiological fouling on the membrane surface remained limited by increased shear forces. In contrast, the decrease in HPC retention at 0.5 L/min, from 97.36% to 82.73%, confirms the progressive development of biological fouling under conditions of reduced convective mass transfer.

At the same time, COD retention rates exceeding 90% at 3 L/min demonstrate that the accumulation of oxidizable organic compounds on the membrane surface remained limited under conditions of high hydrodynamic shear.

The reduction in BOD_5_ and TOC retention in cycles C2 and C3 confirms the progressive accumulation of the biodegradable and dissolved organic fractions in the boundary layer, with a direct effect on the resistance to the transport of organic compounds and the development of organic fouling.

The results demonstrate that the hydrodynamic regime simultaneously controls the transport of dissolved species, concentration polarization, and the accumulation of organic and biological fouling during the cyclic operation of the PA-RO membrane.

### 3.6. Evaluation of the Effectiveness of the Cleaning Process

The efficiency of the cleaning process was evaluated by determining the reversible and irreversible hydraulic resistance of the PA membrane. Reversible and irreversible fouling resistances were determined for the three filtration cycles, allowing for the evolution of membrane fouling over time to be observed.

The irreversible resistance in the first cycle was not determined because the membrane was not subjected to the cleaning process; therefore, it was no longer possible to determine the flux after cleaning. Consequently, only the reversible resistance was determined.

**Figure 11:**
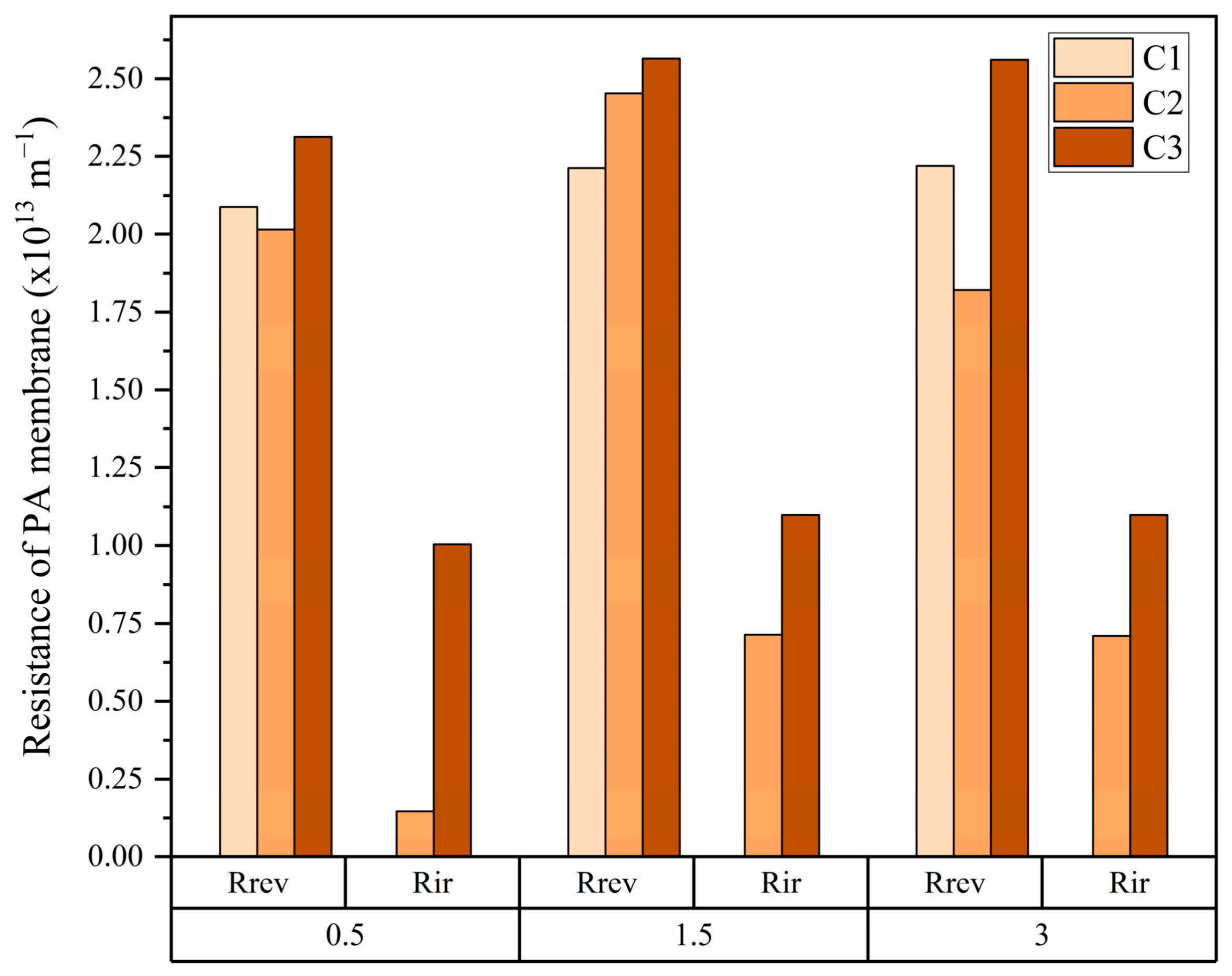
Average variation in reversible and irreversible resistance (Rrev and Rir) determined for each filtration and washing cycle at a pressure of 6 bar, tested at flow rates of 0.5, 1.5, and 3 L/min.

Figure 11 illustrates the average variation in reversible and irreversible resistance determined after each filtration and chemical cleaning at a pressure of 6 bar with a flow rate of 0.5 L/min.

The dominant values of the reversible resistance (Rrev) compared to the irreversible resistance (Rir) indicate that the predominant fouling mechanism was associated with the accumulation of deposits on the surface of the PA-RO membrane and concentration polarization phenomena. The progressive increase in Rrev between successive cycles highlights the gradual development of the superficial fouling layer and the increase in hydraulic resistance during the cyclic filtration of the real effluent from the slaughterhouse.

At the flow rate of 0.5 L/min, the lowest values were obtained for both Rrev and Rir, indicating a slower and more stable development of the clogging mechanisms compared to the other conditions investigated. The low variations in Rrev between cycles C1 and C2 suggest that the deposits formed during the filtration remained predominantly superficial and were partially removed by CP. The increase in both components in cycle C3 highlights the gradual accumulation of residual foulant and the increase in the hydraulic resistance during the successive operation.

At 1.5 L/min, the continuous increase in both Rrev and Rir between successive cycles indicates the progressive accumulation of deposits and the development of a persistent fouling component that could not be completely eliminated by the application of CP. Compared to 0.5 L/min, the more pronounced increase in the irreversible component suggests a reduction in the efficiency of membrane permeability recovery between successive filtration cycles.

In the case of the 3 L/min flow rate, the temporary reduction in Rrev in cycle C2 indicates the partial recovery of permeability after the application of CP and the removal of a significant fraction of the superficial deposits. However, the sharp increase in Rrev and Rir values in cycle C3 highlights the rapid formation of a new fouling layer and the progressive accumulation of a persistent foulant fraction during the successive filtration. This behavior suggests that, at high cross-flow velocities, the accumulation of deposits during filtration exceeded the membrane recovery capacity after CP.

The lower values of Rir compared to Rrev show that persistent severe fouling was not the dominant clogging mechanism in the analyzed experimental interval. However, the gradual increase in the irreversible component between successive cycles confirms the accumulation of organic and inorganic fractions that could not be completely removed by the applied cleaning protocol.

Overall, the analysis of hydraulic resistances highlights that the performance of the PA-RO membrane was predominantly controlled by the development of superficial fouling and the progressive accumulation of the irreversible component during the cyclic filtration of the slaughterhouse effluent. In the case of real slaughterhouse effluents, the effect of reducing fouling by increasing the cross-flow velocity is limited and does not manifest proportionally, as is frequently reported for model systems or simple effluents. The results indicate that, under these conditions, hydrodynamic intensification simultaneously leads to an increase in shear forces and to an amplification of convective transport of contaminants, favoring the formation of more compact and persistent deposits and, implicitly, an increase in the irreversible component during cyclic operation.

### 3.7. Performance and Clogging Assessment of the Membrane

**Figure 12:**
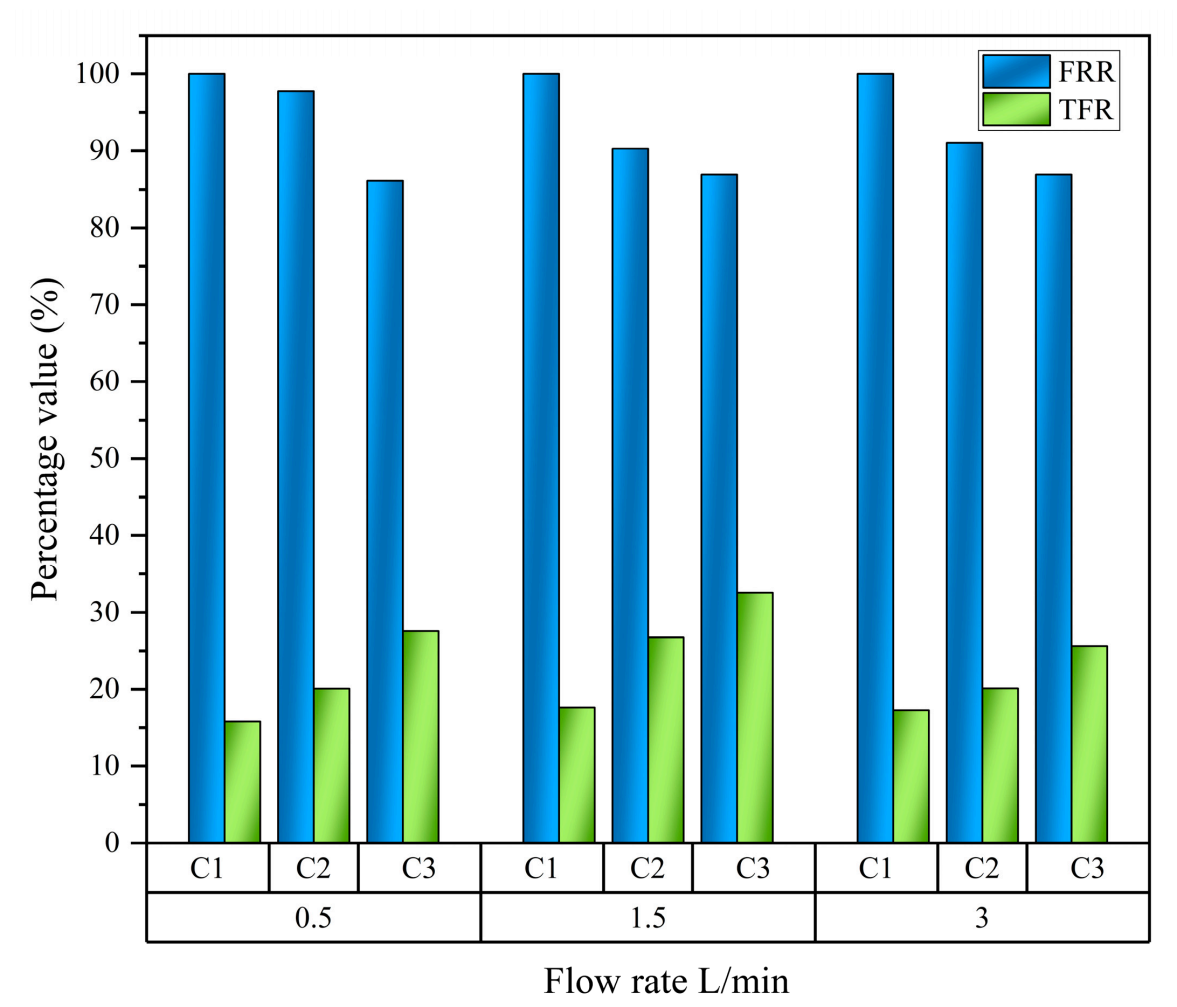
Average percentage variation in FRR and TFR values determined after filtration and cleaning cycles tested at the three flow rates with a constant pressure of 6 bar of the PA and RO membranes.

Figure 12 shows the average percentage variation in FRR and TFR determined after the filtration and cleaning cycles, which were tested at different operating flow rates of 0.5, 1.5, and 3 L/min at a pressure of 6 bar.

As can be seen in the graph, the average variation in FRR is inversely proportional to the average variation in TFR. This trend is normal, since FRR shows the degree of recovery of the permeate flux after the cleaning process, and TFR shows as percentage values how much of the permeate flow was lost after the filtration process. In the case of TFR, the values increase progressively regardless of the filtration cycle.

The FRR and TFR values determined at 6 bar highlight the progressive degradation of the hydraulic performance of the PA-RO membrane during the cyclic filtration of real slaughterhouse wastewater. The decrease in FRR from 100% in C1 to approximately 86–87% in cycle C3 confirms the gradual reduction in the membrane’s ability to recover its initial flux after CP application, indicating the progressive accumulation of a persistent fraction of organic pollutant compounds and microorganisms between successive cycles.

The simultaneous increase in TFR highlights the increase in total flux loss during repeated operation. The most pronounced increase was observed at 1.5 L/min, where TFR increased from 17.6% to 32.58%, indicating the highest cumulative fouling accumulation among the investigated conditions. In contrast, at 0.5 L/min, TFR increased moderately to 27.57%, and FRR values remained the highest, indicating the best hydraulic recovery of the membrane.

The results are relevant because at 0.5 L/min, although the effective pressure maintained in the membrane module was higher due to lower hydraulic losses, the membrane presented the best operational stability and the lowest cumulative flux loss. These results show that, under the investigated conditions, increasing the cross-flow rate to 1.5 and 3 L/min did not improve the membrane permeability recovery after cleaning and was associated with higher TFR values and with a progressive decrease in FRR between successive filtration cycles.

## 4. Statistical Analysis

Experimental investigation of membrane processes applied to real industrial effluents involves complex interactions between operational parameters (e.g., flow rate, operating time) and response variables that describe both membrane performance and water quality (e.g., permeate flux, turbidity, conductivity, pH).

Both statistical analysis and mathematical modeling were applied only to the operational parameters of the RO process and to the quality indicators monitored during the experiments. In contrast, detailed statistical analyses were not applied for the physicochemical or biological quality indicators, as they were determined before and after the RO process.

Under these conditions, classical descriptive analysis is not sufficient to fully highlight the relationships between variables. Therefore, the use of advanced statistical methods becomes essential for identifying patterns, correlations, and latent structures in the experimental data.

The application of multivariate statistical analysis techniques in the field of membrane processes and wastewater treatment is widely documented in the specialized literature. These methods allow for the reduction in data dimensionality, the identification of dominant factors influencing the process, as well as the grouping of variables according to their similarity or functional behavior. In particular, cluster analysis and correlation-based methods have proven effective in separating input variables, water quality indicators, and performance parameters within complex treatment systems.

In the case of RO processes, where fouling phenomena, hydrodynamic conditions, and pollutant characteristics act simultaneously on the behavior of the system, the use of statistical tools becomes indispensable for the interpretation of experimental results.

Multivariate data analysis is widely used to identify operational parameters that control the performance of membrane processes and to develop predictive models. Numerous studies have demonstrated that techniques such as HCA and PCA allow for the grouping of variables with similar behavior, reducing the complexity of data sets and highlighting the dominant factors of the process. At the same time, they have shown that the integration of statistical analysis with mathematical modeling allows for the description and prediction of the development of fouling phenomena. In line with these approaches, the methodology applied in the present study uses HCA to identify functional groups of parameters and develop mathematical models capable of describing the evolution of permeate flux, retention efficiency and fouling mechanisms in the reverse osmosis process [56,57].

At the same time, the analysis of statistical correlations represents a solid basis for the subsequent formulation of mathematical models. By identifying stable relationships between input variables (such as time and flow rate) and output parameters (permeate volume and quality indicators), it becomes possible to build generalized functional dependencies that describe the behavior of the system under different operating conditions. This approach is particularly relevant in the case of cyclic processes, characterized by a nonlinear evolution of membrane performance and simultaneously influenced by reversible and irreversible mechanisms.

Therefore, the main objective of the statistical analysis chapter is to identify and quantify the correlations between operational parameters and experimentally obtained variables in order to:

- Classify the studied parameters into homogeneous functional groups;
- Highlight the dominant control parameters of the system;
- Establish a coherent framework for the development of mathematical models specific to the membrane behavior and the quality of the treated effluent.

The results of this analysis provide a solid theoretical and quantitative basis for the subsequent mathematical modeling stage, ensuring that the proposed relationships are both statistically significant and physically relevant in the context of wastewater treatment processes using membrane technologies.

In the context of complex experimental analyses, which involve a high number of interdependent variables, multivariate statistical methods become essential for understanding the internal structure of the data. One of the most widely used techniques for this purpose is Hierarchical Cluster Analysis (HCA), an exploratory method that allows for the identification of similarity relationships between variables or observations and their organization into homogeneous groups (clusters), based on distance and similarity criteria.

HCA offers a suggestive graphical representation in the form of dendrograms, through which the hierarchical links between the analyzed parameters can be highlighted. Thus, variables that show similar behaviors from a statistical point of view are grouped together, indicating the existence of common physical or chemical mechanisms that govern their evolution. In the case of membrane processes, this approach allows for the separation of input parameters (operational parameters) from output parameters (performance and quality parameters), as well as the identification of relevant functional subgroups.

The importance of using HCA is particularly high in studies targeting nonlinear and dynamic processes, such as RO, where the behavior of the system is simultaneously controlled by hydrodynamic conditions, effluent composition and the evolution of fouling phenomena. By highlighting stable clusters and relationships between variables, HCA allows for the following:

- Identification of dominant variables that control the process;
- Separation of parameters into homogeneous functional groups (e.g.: membrane performance parameters, water quality parameters, dynamic parameters);
- Highlighting variables that exhibit time-dependent or hydrodynamic regime behavior.

An essential aspect of this method is that the results obtained are not only descriptive, but also provide a solid basis for subsequent stages of mathematical modeling. Thus, grouping variables according to their behavior allows for the definition of common functional models for parameters in the same cluster. For example, the identification of a stable cluster formed by the membrane performance parameters indicates the possibility of modeling them through the same mathematical dependence in relation to the input variables.

In this sense, HCA contributes to reducing the complexity of the modeling process by:

- Limiting the number of mathematical relationships required;
- Substantiating the choice of independent and dependent variables;
- Increasing the physical relevance and robustness of the proposed models.

To perform the HCA in this study, the OriginLab program, version 2019, was used, which offers dedicated tools for multivariate statistical analysis and dendrogram generation. The choice of this software was determined by its ability to process complex experimental data sets, apply different distance metrics and linkage methods, and provide clear, easy-to-interpret graphical representations.

Therefore, the use of hierarchical cluster analysis represents an essential step in understanding the mechanisms that govern the studied process and in establishing a coherent direction for the development of mathematical models specific to the experimental data obtained.

The results of the Hierarchical Cluster Analysis (HCA) analysis are presented in the form of dendrograms, which provide a synthetic image of the similarity relationships between the analyzed parameters. These graphical representations allow us to highlight the hierarchical structure of the data and identify the natural groupings of variables, depending on their statistical behavior.

The interpretation of dendrograms plays an essential role in understanding the mechanisms governing the studied process, as it allows for the delimitation of clusters corresponding to different types of parameters, such as input variables (operational), water quality indicators, and specific membrane performance parameters. At the same time, the analysis of their grouping provides information on the dependencies between variables, the stability of the identified relationships, and the influence of operating conditions on the system.

Therefore, the interpretation of HCA results represents an essential intermediate step between exploratory statistical analysis and the development of mathematical models, facilitating the identification of relevant functional relationships and substantiating the choice of model structure used to describe experimental data.

**Figure 13:**
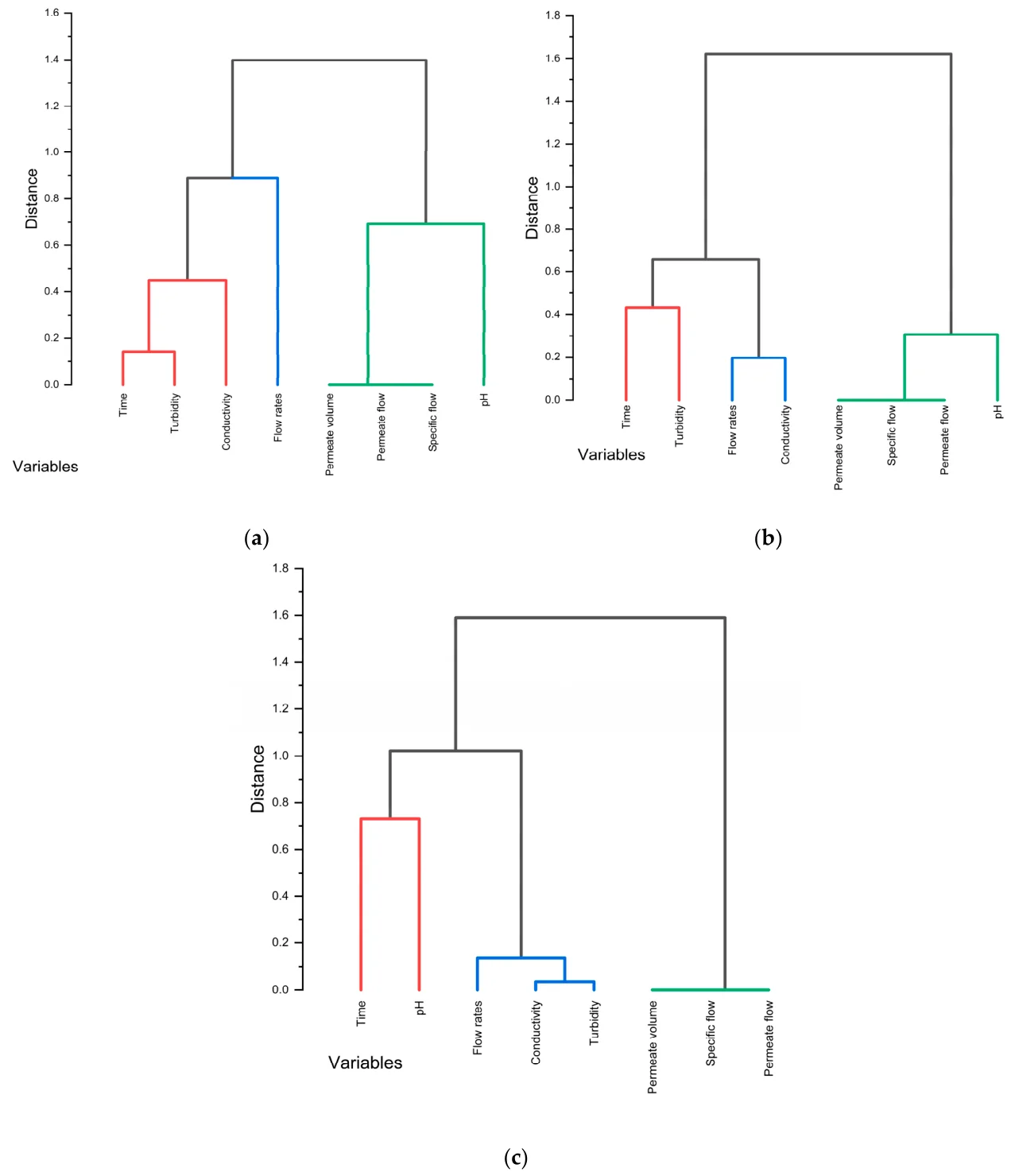
Representation of specific dendrograms: (**a**) C1; (**b**) C2; (**c**) C3.

The results of the statistical analysis, specific to each cycle, are presented in Figure 13.

Following the analysis of the dendrograms in Figure 13, the following conclusions can be drawn:

The dendrogram for C1 highlights the existence of three main clusters, which reflect the functional structure of the analyzed system. The first cluster groups the experiment time, turbidity, and conductivity, suggesting a direct correlation between the evolution of the process and the change in water quality parameters. The second cluster is represented by the concentrated flow rate (Q), which appears separately, confirming its role as an independent input variable and a dominant control parameter. The third cluster includes the permeate volume, membrane flux, specific flux, and pH, indicating a strong correlation between the parameters that characterize the membrane’s performance. Overall, the obtained structure highlights the clear separation between the dynamic parameters, the input variable and the response parameters, which constitutes an important basis for defining mathematical models, in which the input variables (time and flow rate) control the evolution of the membrane performance parameters.

The dendrogram corresponding to C2 highlights a reorganization of the structure of the relationships between variables, compared to the previous cycle, which reflects an evolution of the mechanisms that control the filtration process. It is observed that the experiment time and pH are grouped together, which indicates that, at this stage, the pH acquires a dynamic character, being directly influenced by the evolution of the process over time. This association suggests a change in the chemical equilibrium of the system during the membrane’s operation. At the same time, the concentrated flow rate is found in the same cluster as the conductivity and turbidity, which indicates a significant proximity between the input parameter and the water quality indicators. This distribution highlights the fact that, in C2, the hydrodynamic regime exerted by the flow rate becomes a determining factor in controlling the effluent characteristics. On the other hand, the membrane performance parameters (permeate volume, membrane flux and specific flux) remain compactly grouped, which confirms the stability of the relationship between them and their common role in describing the hydrodynamic behavior of the membrane. Overall, this configuration indicates a transition from a system dominated by time evolution to one in which the flow rate begins to directly influence both water quality and, indirectly, the process performance, an essential aspect for defining the mathematical relationships between the analyzed variables.

The dendrogram corresponding to C3 highlights a relatively stable structure, but with some changes in the way the variables are associated, which indicates the maturation of the phenomena taking place in the system. Three main groups are distinguished. The first cluster brings together the experiment time and turbidity, suggesting the maintenance of a direct link between the process evolution and the variation in the colloidal load, which confirms the dynamic nature of these parameters. The second cluster includes the concentrated flow rate and conductivity, highlighting a clearer correlation between the input parameter and the ionic content of the effluent. This association indicates that, in C3, the influence of the flow rate on the quality characteristics becomes better defined and more stable. The third cluster groups the permeate volume, the membrane flux, and the specific flux, to which pH is also added, suggesting that, at this stage, pH is more strongly correlated with the membrane performance than with the process dynamics. The close relationship between the flow parameters confirms the consistency of this cluster as a descriptor of the hydrodynamic behavior of the membrane.

The hierarchical cluster analysis performed for the three cycles highlights both common elements and changes in the relationships between variables, reflecting the evolution of the filtration process over time.

In the case of the first cycle, the dendrogram indicates a clear separation of the system into three components:

(time, turbidity, conductivity) ∣(flow) ∣(flows, permeate volume, pH)

The existence of a dynamic cluster (time + quality indicators), a distinct control parameter (flow rate), and a membrane performance cluster is observed. This distribution indicates that, in the initial phase, the system is predominantly controlled by time evolution.

For C2, the structure changes as follows:

(time, pH) ∣(flow, conductivity, turbidity) ∣(flows, permeate volume)

The migration of conductivity and turbidity towards the flow-controlled cluster is observed, which highlights an increased influence of hydrodynamic conditions on water quality. At the same time, pH becomes a dynamic, time-dependent variable.

In C3, the system acquires a more stable organization:

(time, turbidity) ∣(flow, conductivity) ∣(flows, permeate volume, pH)

The strengthening of the relationship between flow rate and quality parameters is observed, as is the integration of pH into the membrane performance cluster, which suggests a stronger influence of the separation process on pH.

The comparative analysis of the three cycles allows us to highlight some essential aspects:

- There is a stable cluster of membrane performance parameters (permeate volume, membrane flux, specific flux) present in all cycles, which indicates that these parameters must be treated as a unit in the modeling;
- The concentrated flow rate (Q) remains the dominant control variable, progressively influencing the quality parameters (conductivity, turbidity), an aspect confirmed by their integration into the same cluster in C2 and C3;
- The dynamic cluster (based on time) undergoes variations, including different parameters (turbidity, conductivity, or pH), which shows that these variables are dependent on the process evolution and operating conditions.

The results obtained through HCA represent an essential step in the substantiation of the mathematical modeling process of experimental data. Thus,

- The choice of the main input variables is confirmed:

t(time) and Q (flow))

Output parameters can be grouped according to their statistical behavior, resulting in distinct functional groups, such as:

- Group of performance parameters (volume, fluxes–membrane flux, specific flux);
- Group of quality parameters (conductivity, turbidity);
- Parameters with variable behavior (pH).

This grouping justifies the subsequent modeling approach in which, using the TableCurve 3D program version 4.0.05, sets of mathematical equations are generated for each parameter.

### Identification of Mathematical Models

Based on the results obtained in the HCA statistical analysis, which allowed us to highlight the relationships between the studied variables and group them according to their behavior, the mathematical modeling stage of the experimental data was initiated. This stage has the role of quantitatively describing the dependence between the input and output parameters, as well as identifying the functional relationships representative of the behavior of the analyzed system.

The cluster analysis highlighted the fact that the studied parameters can be grouped according to their physical nature and the identified statistical correlations, thus resulting in distinct functional groups of the output parameters. This structuring allowed for the definition of a modeling strategy in which the variables are not treated individually, but are grouped into homogeneous categories, which led to a reduction in complexity and an increase in the physical relevance of the obtained models.

In order to identify mathematical relationships, the TableCurve 3D program v. 4.0 was used, which allowed us to generate a large number of candidate equations to describe the dependencies between the input variables (experiment time and concentrated flow rate) and the analyzed output parameters. This software offers the possibility of fitting the experimental data to an extensive variety of functional forms, facilitating the selection of the most appropriate models based on statistical criteria [58,59].

For each parameter studied and for each cycle, extensive sets of mathematical equations were generated, characterized by different levels of accuracy. Subsequently, these equations were subjected to a selection and filtering process based on the equation number (specific number of the TableCurve 3D program) and, subsequently, on the value of the coefficient of determination (R^2^) in order to eliminate irrelevant or weakly correlated relationships [58,59].

This approach led to the definition of generalized mathematical models, characterized by structural consistency and extensive applicability, facilitating both the interpretation of the physical phenomena involved and the practical use of the relationships obtained in the analysis and optimization of the process.

**Figure 14:**
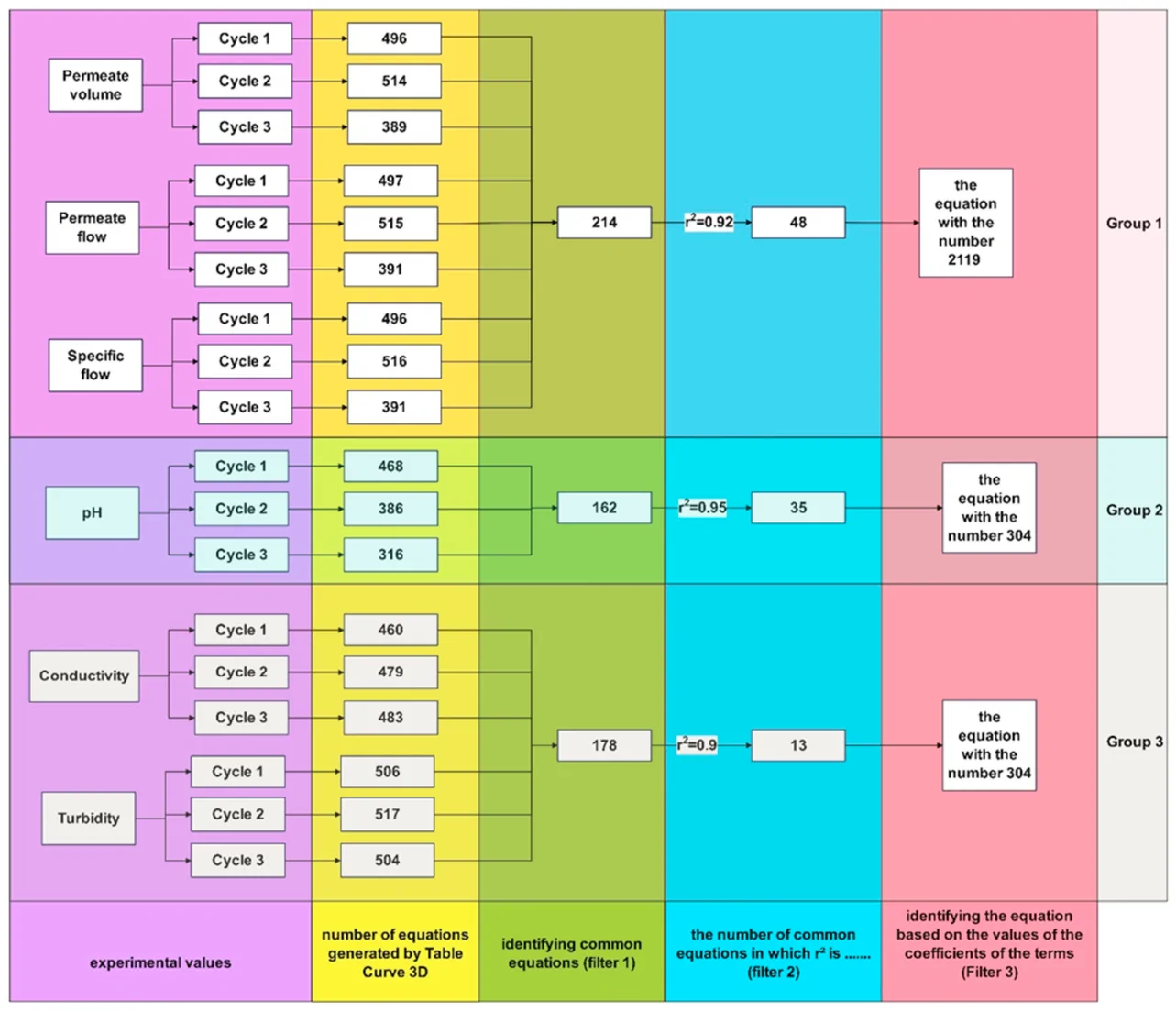
Mathematical equation filtering mode.

The following provides an explanation of what was obtained at each work stage (according to Figure 14) [60,61]:

- Stage 1: For each work cycle, sets of equations specific to each cycle were generated as follows in the table.
- Stage 2: Taking into account the grouping method of the studied parameters obtained following the statistical analysis (presented above), the following were identified:
- For group 1, a number of 214 common equations;
- For group 2, a number of 162 common equations;
- For group 3, a number of 178 common equations.
- Stage 3: From these equations, an analysis was carried out on their value of the correlation coefficient r2 and for a value of 0.9 for each individual group, the following were identified:
- For group 1, a number of 118 equations;
- For group 2, a number of 91 equations;
- For group 3, a number of 13 equations.

Due to the large number of equations identified in the first two groups, only for these groups was the value of the correlation coefficient increased, as follows:

- For group 1, for r^2^ = 0.92, 48 equations are identified;
- For group 2, for r^2^ = 0.95, 35 equations are identified.

For a better understanding of the working methodology, the graphic representation presented in Figure 14 was made. It should also be noted that, during the equation identification stage, an essential role was played by the equation generation program TableCurve 3D, which has a database containing multiple types of equations, each of which was assigned a specific numerical code. This way of organization allowed for the first two stages of filtering the equations to be carried out in an efficient and systematic way.

Even though a set of candidate mathematical models resulted from the filtering system applied to the equations generated using the TableCurve 3D program, these were not considered directly valid, but were subjected to an additional stage of analysis. In this regard, a detailed visual evaluation of the equations was carried out, which had as its objective the analysis of the coefficients associated with each term in the identified mathematical relations.

This stage allowed for the verification of the coherence and consistency of the models by examining the values of the coefficients and the contribution of each term to the final form of the equation. Such an analysis is essential for the practical validation of the models, ensuring that they not only provide a good statistical adjustment, but also present a stable and interpretable mathematical structure.

Following this evaluation, the equations considered representative for each category of parameters were selected. Thus, Table 3 presents the mathematical models identified for each analyzed group and Table 4 summarizes the values of the coefficients related to the terms corresponding to these equations.

Based on the filtering and selection procedure, two mathematical model structures were identified as the most suitable for describing the experimental data.

For Group 1 parameters (permeate volume, permeate flux and specific flux), the selected model is represented by Equation (8):

(8) $$z=\frac{a}{1+e^{\left[-\left(\frac{x-b}{c}\right)\right]}}+\frac{d}{1+e^{\left[-\left(\frac{y-e}{f}\right)\right]}}+g\cdot \frac{1}{1+e^{\left[-\left(\frac{x-b}{c}\right)\right]}}\cdot \frac{1}{1+e^{\left[-\left(\frac{y-e}{f}\right)\right]}}$$

where z represents the predicted response variable, x is the filtration time, y is the recirculation flow rate, and a–g are the model coefficients.

For Group 2 (pH) and Group 3 (conductivity and turbidity), the selected model is represented by Equation (9):

z = a + bx + clny + dx^2^ + e(lny)^2^ + fxlny (9)

where z is the predicted parameter, x represents filtration time, y corresponds to the recirculation flow rate, and a–f are model coefficients.

The coefficients corresponding to each parameter and filtration cycle are presented in Table 5.

**Figure 15:**
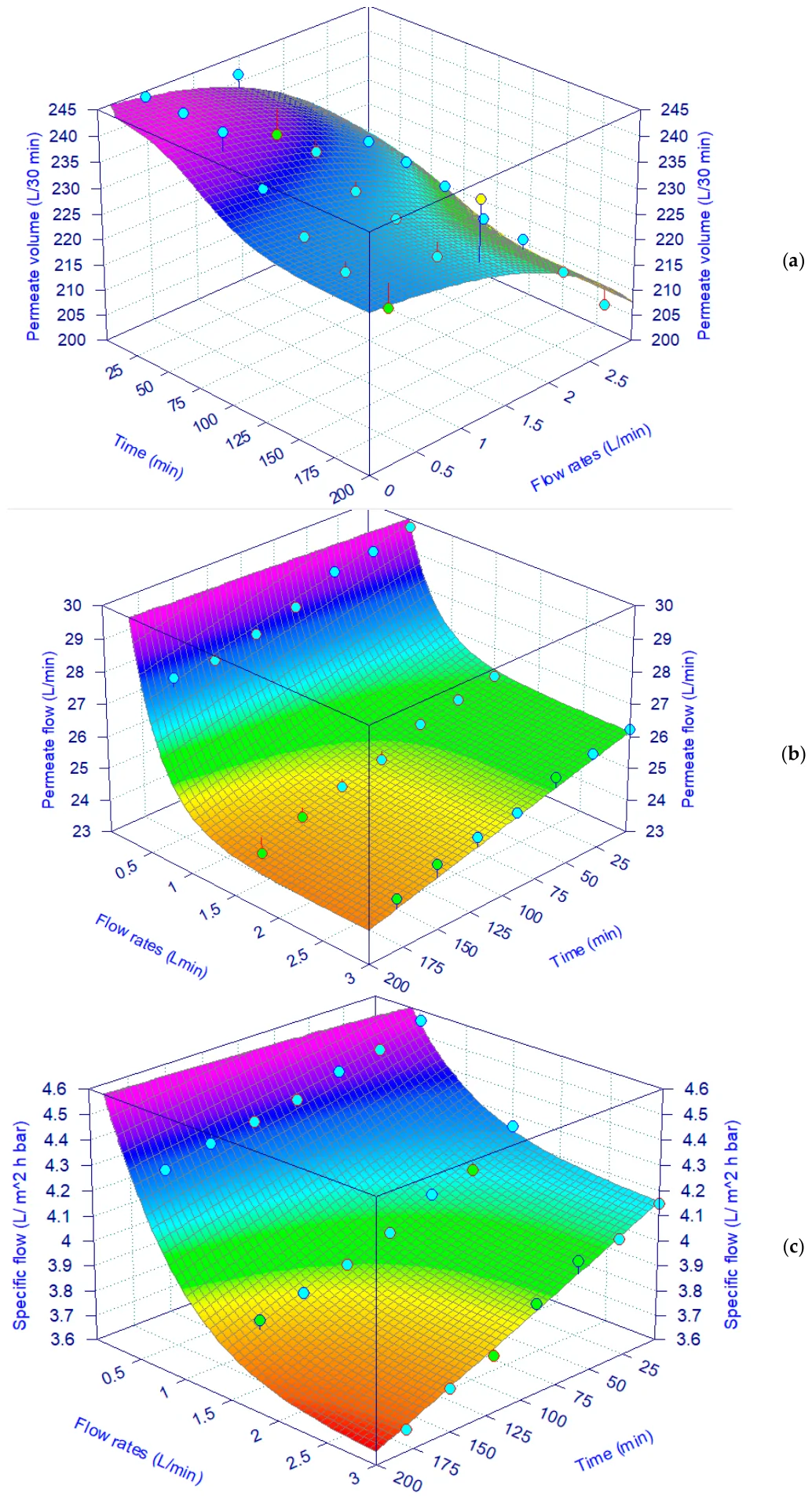

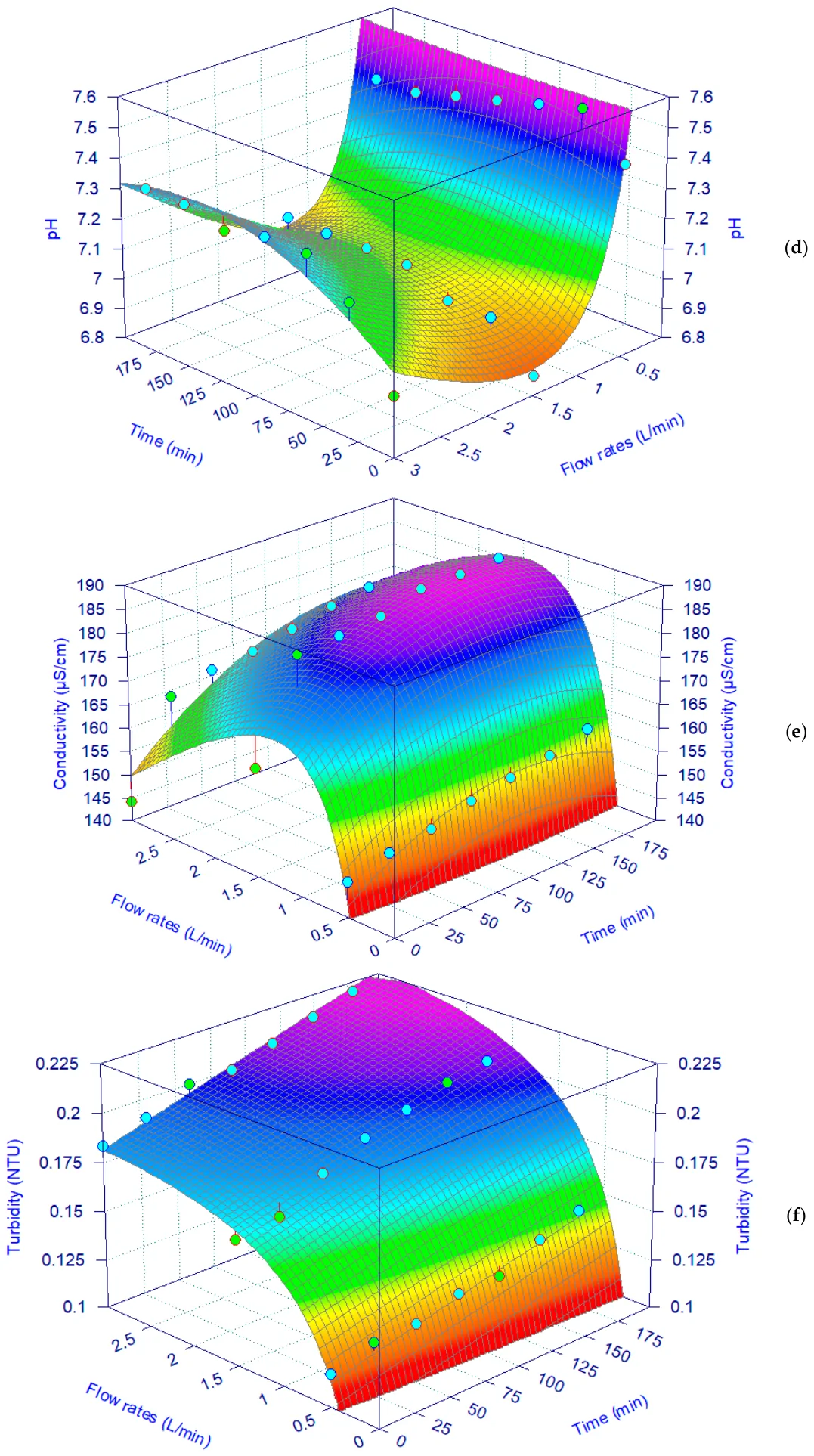
Representation of the surfaces generated by the joint equations specifically identified for the parameter: (**a**) Permeate volume—C1. (**b**) Permeate flow—C2. (**c**) Specific flow—C3. (**d**) pH—C2. (**e**) Conductivity—C1. (**f**) Turbidity—C3.

In Figure 15a–f are illustrated the graphical representations generated using the TableCurve 3D application, which are specific representations of the identified equation. These graphical representations were obtained directly using the TableCurve 3D program.

## 5. Discussion

The evolution of polyamide (PA) membrane performance over three successive filtration cycles highlights a progressive transition from reversible surface fouling to cumulative irreversible fouling, driven by the organic and microbiological composition of the slaughterhouse effluent after the DAF stage, consistent with the multiphase fouling behavior reported for polyamide RO membranes used to treat complex organic effluents [37,38].

The higher flux observed at 0.5 L/min in pure water tests is associated with a more uniform transmembrane pressure distribution, whereas at high flow rates, pressure losses reduce the effective TMP, limiting the flux increase.

At 0.5 L/min, the reduced recirculation rate decreases the mass transfer coefficient in the concentration polarization model, increasing the accumulation of solutes at the membrane’s surface and accelerating the formation of the deposition layer. The predominantly reversible character of fouling in C1, confirmed by low values of irreversible resistance and good flux recovery after cleaning with NaOH/EDTA, is consistent with the surface fouling behavior reported for similar organic loading conditions [38].

The progressive divergence between flux recovery and retention in cycles C2 and C3 represents the central mechanistic result of the study. Flux partially recovers after each cleaning, while the retention of organic and ionic compounds continues to decrease, indicating the simultaneous existence of two distinct fouling mechanisms. Organic fouling involves both surface adsorption and internal sorption of dissolved organic matter, and the irreversible loss of performance is determined by molecular-level interactions between low-molecular-weight organic compounds and the membrane’s active layer. The adsorption of these fractions on the polyamide amide groups (-CO-NH-) modifies the surface charge density and solvate partition coefficients, reducing retention independently of hydraulic permeability. Autopsy studies confirm the presence of polysaccharides, organic sulfonic acids and compounds with carboxylate and amide groups as the main fouling constituents, demonstrating the high chemical affinity between the organic fractions of the effluent and the PA active layer. The progressive increase in the conductivity and pH of the permeate reflects this change in the electrostatic properties of the membrane. This behavior is consistent with the findings of Fatima et al. [33], who reported TDS values of 325 ± 20.41 mg L^−1^ using a flat-sheet PA-TFC membrane to treat wastewater from a poultry slaughterhouse. Although their system operated at a higher pressure (15.16 bar), the comparison indicates that operating pressure alone does not determine ion rejection; it is also influenced by membrane characteristics, feed stream composition, and hydrodynamic conditions.

At 3 L/min, increased shear reduces surface deposits, but high values of Rir indicate the presence of irreversible mechanisms. This apparent contradiction is explained by the non-uniform distribution of TMP along the feed channel. Pressure losses create local areas of high flow, where deposits are mechanically compacted under the effect of hydrodynamic stress, becoming more resistant to chemical cleaning than those formed uniformly at lower speeds. This effect confirms the role of hydrodynamics in the control of fouling in planar modules [37].

Although higher cross-flow velocities are generally expected to reduce concentration polarization and mitigate the accumulation of loosely attached foulants, the present results indicate that this beneficial effect was insufficient to improve the long-term hydraulic performance of the RO membrane treating poultry slaughterhouse wastewater. Similar reductions in ion retention caused by fouling were also reported by Fatima et al. [33], whereas Coskun et al. [55] demonstrated that the high ionic strength of wastewater from poultry slaughterhouses accelerates concentration polarization during prolonged membrane operation. These observations are consistent with the progressive increase in permeate conductivity observed in the present study. The complex composition of the feed, containing dissolved organic matter, colloids, microorganisms, and inorganic species, promoted the development of persistent fouling layers during cyclic operation. Under these conditions, the beneficial effect of increased hydrodynamic shear was likely counterbalanced by the continuous delivery of foulants to the membrane surface, resulting in a progressive increase in irreversible hydraulic resistance.

The progressive decrease in HPC retention at 0.5 L/min, compared to stable values (>99%) at 1.5 and 3 L/min, reflects shear-dependent bacterial adhesion kinetics. These observations are consistent with those of Al-Ashhab et al. [56], who demonstrated that higher cross-flow velocities reduce bacterial adhesion and biofilm formation by increasing the hydrodynamic shear stress at the membrane’s surface. At low velocities, the residence time of bacteria on the surface increases, favoring irreversible attachment and biofilm consolidation. EPS polysaccharides contribute to increasing hydraulic resistance by forming a gel layer and simultaneously reducing flux and retention. The persistence of biological fouling after cleaning is explained by the resistance of the EPS matrix, which protects microorganisms and allows for rapid biofilm recovery. High HPC retention values at higher flow rates confirm the role of shear in limiting bacterial adhesion [47].

The gradual increase in COD concentration in the permeate over successive filtration cycles indicates a gradual decline in membrane selectivity, caused by the accumulation of organic impurities and the incomplete recovery of membrane performance following chemical cleaning. Similar reductions in COD retention, caused by fouling, were reported by Fatima et al. [44], who demonstrated that chemical cleaning only partially restored membrane permeability, as residual organic matter remained attached to the membrane surface after repeated filtration cycles. Compared to their results, the higher initial COD rejection rate obtained in the present study suggests that the selected hydrodynamic conditions delayed the development of fouling during the early stages of cyclic operation. Similarly, Coskun et al. [55] reported COD removal efficiencies of approximately 78% using flat-sheet PA membranes operated at 5 bar, which are comparable to the COD retention achieved in the present study.

The evolution of the COD/BOD_5_ and COD/TOC ratios confirms the selective retention of macromolecular compounds in C1, followed by the progressive loss of selectivity in C2 and C3, associated with irreversible pore modification and surface chemical changes. In a study conducted by Maizatul Azrina Yaakob et al., the COD/BOD_5_ ratio was analyzed for wastewater from a poultry slaughterhouse, whose COD (5422.25 ± 2282.69 mg O2/L) and BOD_5_ (1602 ± 242.7 mg/L) concentrations were much higher than the concentrations found in the present study. This is also confirmed by the increased COD/BOD_5_ ratio with average values of 2.0–2.5. This ratio increases the degradation time of organic matter [61]. In general, the organic matter in the actual effluent comes from the presence of a mixture of proteins, fibers, fats, and blood. This decoupling between selectivity and permeability is characteristic of fouling caused by low molecular weight organic compounds in polyamide membranes [62], which cannot be completely removed by standard alkaline cleaning.

Overall, the data support a three-step fouling progression model: reversible surface deposition and hydrodynamically controlled concentration polarization in C1; adsorption of low molecular weight organic fractions on the polyamide functional groups, combined with the initial consolidation of the biofilm in C2, leading to flux–retention divergence; and dominant irreversible pore blocking, associated with the stabilization of the EPS matrix in C3, where cleaning with NaOH/EDTA only allows for partial flux recovery without restoring selectivity. This mechanistic framework, supported by the quantification of resistances for different cycles and flow rates, contributes to the understanding of the behavior of PA membranes under real industrial effluent conditions and provides a basis for optimizing pretreatment and cleaning strategies, adapted to the identified fouling mechanisms. From a practical standpoint, the gradual increase in irreversible fouling and the decrease in flow recovery observed during cyclic operation indicate that additional pretreatment strategies warrant investigation to improve the long-term performance of reverse osmosis systems designed for the treatment of wastewater from poultry slaughterhouses. Although ultrafiltration (UF) is commonly used upstream of RO in industrial applications, the evaluation of this configuration was not the focus of this study. Future research should compare the DAF–RO and DAF–UF–RO configurations in terms of fouling trends, hydraulic resistance development, membrane stability, operating costs, and overall economic feasibility.

A limitation of the present study is that membrane characterization techniques such as SEM, FTIR, AFM, or XPS were not performed. Consequently, the proposed mechanisms are interpreted from the evolution of hydraulic resistances, flux recovery, and separation performance rather than from direct structural evidence of the membrane. Under these conditions, the observed decline in performance is consistent with the progressive accumulation of residual foulants and concentration polarization during cyclic operation. Future work should combine cyclic RO experiments with membrane surface characterization to directly identify the composition and distribution of residual foulants and further validate the mechanisms proposed in this study.

## 6. Conclusions

Cyclic evaluation of the polyamide RO membrane (PA) demonstrated that the long-term performance of the process is mainly controlled by the evolution of fouling phenomena and cannot be characterized exclusively by the instantaneous retention efficiency. An integrated analysis of permeate flux, Flux Recovery Rate (FRR), reversible (Rrev), and irreversible (Rir) hydraulic resistances, together with the retention efficiency of physicochemical and microbiological indicators, allowed for a comprehensive evaluation of the membrane behavior during successive filtration and chemical cleaning cycles.

Among the operating conditions analyzed, the recirculation flow rate of 0.5 L min at 6 bar provided the greatest hydraulic stability, as reflected by the most stable flux recovery and the lowest resistance to hydraulic fouling. However, microbiological retention showed a different trend than the hydraulic indicators during cyclic operation, demonstrating that hydraulic and microbiological performance were not optimized under the same operating conditions. The evolution of the ratios between chemical oxygen demand and biochemical oxygen demand (COD/BOD_5_), biochemical oxygen demand and total organic carbon (BOD_5_/TOC), and chemical oxygen demand and total organic carbon (COD/TOC) highlighted the preferential retention of less biodegradable organic fractions and the modification of the organic matter profile after the reverse osmosis process.

The main contribution of this study consists of the integration of hierarchical clustering analysis (HCA) with mathematical modeling to evaluate membrane performance in cyclic operation. The identification of stable groups of parameters with similar behavior allowed for the development of common predictive models, with high coefficients of determination (R^2^ > 0.90), reducing the complexity of the process description and providing a tool for optimizing the operating conditions and cleaning strategies applied to reverse osmosis processes intended to treat real wastewater from poultry slaughterhouses. The results obtained in this study contribute to a better understanding of the long-term behavior and fouling trends of commercial (PA) RO membranes used to treat wastewater from poultry slaughterhouses. Future research should focus on optimizing RO processes by integrating appropriate pretreatment steps, with the aim of reducing membrane fouling, improving long-term performance, and enhancing the reliability of the wastewater treatment and reuse.

## Figures and Tables

**Table 1:** Characterization of wastewater sampled after the poultry slaughterhouse treatment process.

Variable	Unit of Measurement	Range Value	Average of Total Values
pH	Unit	6.88–7.12	7.00
Conductivity	µS/cm	907–1073	992
Turbidity	NTU	10.46–16.46	13.58
TSS	mg/L	23.6–29.8	26.5
COD	mg O_2_/L	262.1–325.3	294.2
BOD_5_	mg/L	135–179	156
TOC	mg/L	101.3–129.9	115.5
HPC	CFU/mL	53.71–79.67 × 10^5^	66.20 × 10^5^

**Table 2:** Average quality indicator reports.

Operating Conditions	Source of Analysis	BOD_5_/TOC	BOD_5_/COD	COD/BOD_5_	COD/TOC
6 bar 0.5 L/min	Real effluent	1.44	0.56	1.79	2.59
	Permeate C1	1.20	0.89	1.12	1.34
	Permeate C2	1.25	0.94	1.07	1.34
	Permeate C3	1.31	0.87	1.15	1.50
6 bar 1.5 L/min	Real effluent	1.31	0.47	2.14	2.81
	Permeate C1	1.11	0.82	1.21	1.35
	Permeate C2	1.23	0.89	1.12	1.38
	Permeate C3	1.36	0.93	1.07	1.46
6 bar 3 L/min	Real effluent	1.13	0.38	2.61	2.94
	Permeate C1	1.02	0.78	1.29	1.31
	Permeate C2	1.17	0.87	1.15	1.35
	Permeate C3	1.06	0.78	1.29	1.36

**Table 3:** Number of equations generated using the TableCurve 3D program for each parameter studied.

Nr. Crt.	Group	Parameter	Cycle	Number of Equations
1.	Group 1	Permeate volume	1	496
2.			2	514
3.			3	389
4.		Membrane flow	1	497
5.			2	515
6.			3	391
7.		Specific flow	1	496
8.			2	516
9.			3	391
10.	Group 2	pH	1	468
11.			2	386
12.			3	316
13.	Group 3	Conductivity	1	460
14.			2	479
15.			3	483
16.		Turbidity	1	506
17			2	517
18.			3	504

**Table 4:** Selected mathematical models for the analyzed parameter groups and their corresponding coefficients of determination (R^2^).

Nr. Crt.	Group	The Identified Equation	Parameter	Cycle	Correlation Coefficient
1	1	Equation (8)	Permeate volume	1	0.92
2				2	0.98
3				3	0.99
4			Permeate flow	1	0.92
5				2	0.98
6				3	0.99
7			Specific flow	1	0.92
8				2	0.98
9				3	0.99
10	2	Equation (9)	pH	1	0.99
11				2	0.97
12				3	0.99
13	3		Conductivity	1	0.95
14				2	0.98
15				3	0.99
16			Turbidity	1	0.91
17				2	0.92
18				3	0.99

**Table 5:** Presentation of the values corresponding to the coefficients of the terms in the identified equations.

Coefficient	Permeate Volume			Permeate Flow			Specific Flow			pH			Conductivity			Turbidity		
	1	2	3	1	2	3	1	2	3	1	2	3	1	2	3	1	2	3
A	−42.5841918344934	294.361648231853	454.998175806987	−5.30148668963508	36.6463303120888	56.6446530727652	−0.883581114939176	6.107721719	9.440775512	7.02106774133424	6.97377873998402	6.76718482226634	168.136115331748	205.642519970074	251.425546695308	0.101110197908602	0.130442336846245	0.152634992032597
B	78.7147000018778	682.269993981903	218.23632811782	78.7147000018778	682.269993981903	218.23632811782	78.7147000018778	682.269994	−218.2363281	0.00158066879785784	0.00278046682874912	0.00225467017136973	0.235989542826361	0.283722951746773	0.1038872614097	0.000194870113914876	0.00012132901766997	0.000201154956288886
C	−20.0473354333099	−748.062384637943	−898.355176157558	−20.0473354333099	−748.062384637943	−898.355176157558	−20.0473354333098	−748.0623846	−898.3551762	−0.2999560393115	−0.387171399277912	−0.366312989050455	15.1708666657009	35.9690452211672	49.1829238058157	0.00157087604172147	0.0277393120370777	0.0442180346335298
D	232.360620704555	45691652.3681761	563.729634551734	28.9275593780959	5688347.63375989	70.1810936261142	4.82125989634931	948057.939	11.69684894	−0.00000308641975308628	−0.0000133744855967078	−0.00000925925925925924	−0.000835831863609642	−0.000973398001175779	−0.000210170487948266	−0.00000016166960611405	0.00000049970605526161	−0.0000000293944738389187
E	5.41529366358984	−4.3795799324096	−0.305227468634067	5.41529366358985	−4.37957993240959	−0.30522746863411	5.41529366358985	−4.379579932	−0.305227469	0.623094971225426	0.434032926091437	0.319006721349592	−29.0659023950444	−22.32953129828	−19.3429167780583	−0.0113152751213481	−0.0372503729682885	−0.016518008648976
F	−1.13555861169097	−0.332195513543349	−0.599942379846912	−1.13555861169097	−0.332195513543349	−0.599942379846916	−1.13555861169097	−0.332195514	−0.59994238	0.000171341484877788	0.00101767841907514	0.000418240783587876	0.0354379700652318	0.0732817468957951	0.00959720091780207	0.000057146765875831	0.00000134698651944718	0.0000290028138638196
G	58.2547949012004	33666370.4030399	−1076.32281527742	7.25238654232192	4191269.26897475	−133.995993187361	1.20873109038698	698544.8782	−22.33266553									

## Data Availability

The original contributions presented in this study are included in the article. Further inquiries can be directed to the corresponding author.

## Abbreviations

The following abbreviations are used in this manuscript:

BOD_5_	Biochemical Oxygen Demand
C1, C2, C3	Cycles
IE	Industrial Effluent
PP	Polypropylene filter
PE	Purified effluent
P1 and P2	Sampling points
IOP	Invariable operating parameters
VOP	Variable operating parameters
QP	Quality parameters
COD	Chemical Oxygen Demand
CP	Cleaning Protocol
DW	Distilled Water
EDTA	Ethylenediaminetetraacetic Acid
HPC	Heterotrophic Plate Count
PA	Polyamide
R_ir_	Irreversible Resistance
R_rev_	Reversible Resistance
RO	Reverse Osmosis
UF	Ultrafiltration
DAF	Dissolved air flotation
FRR	Flow Recovery Rate
TFR	Total Flow Reduction Ratio
TDS	Total Dissolved Solids
TOC	Total Organic Carbon
k	Conductivity
TSS	Total Suspended Solids
OM	Operational and maintenance
HCA	Hierarchical Cluster Analysis
